# Validation and association of candidate markers for adult migration timing and fitness in Chinook Salmon

**DOI:** 10.1111/eva.13026

**Published:** 2020-06-08

**Authors:** Ilana J. Koch, Shawn R. Narum

**Affiliations:** ^1^ Columbia River Inter‐Tribal Fish Commission Hagerman ID USA

**Keywords:** association testing, fitness, migration timing, salmonid

## Abstract

Recent studies have begun to elucidate the genetic basis for phenotypic traits in salmonid species, but many questions remain before these candidate genes can be directly incorporated into conservation management. In Chinook Salmon (*Oncorhynchus tshawytscha*), a region of major effect for migration timing has been discovered that harbors two adjacent candidate genes (greb1L, rock1), but there has been limited work to examine the association between these genes and migratory phenotypes at the individual, compared to the population, level. To provide a more thorough test of individual phenotypic association within lineages of Chinook Salmon, 33 candidate markers were developed across a 220 Kb region on chromosome 28 previously associated with migration timing. Candidate and neutral markers were genotyped in individuals from representative collections that exhibit phenotypic variation in timing of arrival to spawning grounds from each of three lineages of Chinook Salmon. Association tests confirmed the majority of markers on chromosome 28 were significantly associated with arrival timing and the strongest association was consistently observed for markers within the rock1 gene and the intergenic region between greb1L and rock1. Candidate markers alone explained a wide range of phenotypic variation for Lower Columbia and Interior ocean‐type lineages (29% and 78%, respectively), but less for the Interior stream‐type lineage (5%). Individuals that were heterozygous at markers within or upstream of rock1 had phenotypes that suggested a pattern of dominant inheritance for early arrival across populations. Finally, previously published fitness estimates from the Interior stream‐type lineage enabled tests of association with arrival timing and two candidate markers, which revealed that fish with homozygous mature genotypes had slightly higher fitness than fish with premature genotypes, while heterozygous fish were intermediate. Overall, these results provide additional information for individual‐level genetic variation associated with arrival timing that may assist with conservation management of this species.

## 1 INTRODUCTION

The preservation of genetic and phenotypic diversity remains a key strategy of conservation management (Pellens & Grandcolas, 2016). Unfortunately, anthropogenic pressures are causing a rapid decrease in biodiversity, creating both the threat of local extirpation and species’ extinction (Alberti et al., 2017; Hendry, Farrugia, & Kinnison, 2008; West‐Eberhard, 2003). In the face of rapid environmental challenge and change, genetically based adaptation can serve to mitigate risk of extinction (Dawson, Jackson, House, Prentice, & Mace, 2011; Funk, Forester, Converse, Darst, & Morey, 2019; Nicotra, Beever, Robertson, Hofmann, & O’Leary, 2015). As such, both reproductive isolation and local adaptation are strongly considered in conservation decisions (Waples, 1991).

Anthropogenic pressure in the form of habitat degradation, overfishing, climate change, and dam construction has rendered Pacific salmon (*Oncorhynchus* spp.) extirpated from over one‐third of their historical range (Gustafson et al., 2007; Levin & Schiewe, 2001; Muñoz, Farrell, Heath, & Neff, 2015), and approximately half of the remaining populations are listed as threatened or endangered under the U.S. Endangered Species Act. Variation in migration timing by Chinook Salmon (*Oncorhynchus tshawytscha*) is adaptive and impacted by anthropogenic pressure (McClure et al., 2008; Thompson et al., 2019; Waples, Zabel, Scheuerell, & Sanderson, 2008). Previous research has also suggested that adult migration timing is heritable in salmonids (Carlson & Seamons, 2008; Quinn, McGinnity, & Reed, 2016; Quinn, Unwin, & Kinnison, 2000; Thériault, Garant, Bernatchez, & Dodson, 2007) and is strongly associated with a genomic region of major effect in Pacific salmon spp (Hess, Zendt, Matala, & Narum, 2016; Micheletti, Hess, Zendt, & Narum, 2018; Narum, Di Genova, Micheletti, & Maass, 2018; Prince et al., 2017; Thompson et al., 2019).

Substantial variation exists in the timing of Chinook Salmon migration. Early‐returning populations enter freshwater when they are sexually premature, undergoing maturation while holding in freshwater. Late‐returning populations mature in the ocean, prior to entry into freshwater (Quinn et al., 2016). Variation in migration timing can be divergent both within and across populations (Narum et al., 2018; Narum, Hess, & Matala, 2010; Waples, Teel, Myers, & Marshall, 2004). More specifically, three distinct phylogenetic lineages have been identified which exhibit both neutral and adaptive divergence, including the coastal, Interior ocean‐type, and Interior Columbia River stream‐type lineages (Hecht, Matala, Hess, & Narum, 2015). Both the coastal and Interior ocean‐type lineages exhibit variation in arrival timing for spawning as early (spring or summer) or late (fall) phenotypes that correspond to sexually premature and mature individuals entering freshwater, respectively. In contrast, Chinook Salmon from the Interior stream‐type lineage exclusively enter freshwater as sexually premature, but exhibit variation as early versus late in their final ascent to spawning grounds (Hess, Whiteaker, Fryer, & Narum, 2014; Narum et al., 2018; Quinn et al., 2016; Waples et al., 2004). However, it is uncertain whether early and late arrival to spawning grounds is a phenotypic trait that is conserved across lineages of Chinook Salmon despite differences in freshwater entry (Narum et al., 2018).

Despite these differences in freshwater migration timing and arrival to spawning grounds, previous studies indicate that gene flow occurs between early and late phenotypes within populations (Narum et al., 2018; O’Malley, Jacobson, Kurth, Dill, & Banks, 2013; Prince et al., 2017). However, recent environmental pressures select against the early phenotype and, overall, fish that migrate early are at a greater risk of extirpation (Kareiva, Marvier, & McClure, 2000; Quinn et al., 2016; Quinones, Holyoak, Johnson, & Moyle, 2014; Thompson et al., 2019). In spite of selection favoring the late phenotype under various conditions, the ecological, cultural, and historical importance of the early arriving phenotype remains vital for this species (Quinn et al., 2016; Swezey & Heizer, 1977). As such, maintenance of the two migration phenotypes is an extremely important component of salmonid conservation but application to management decisions based on the genetic architecture of the trait remains a continuing debate (Oke & Hendry, 2019; Pearse, 2016; Quinn et al., 2016; Schindler et al., 2010; Waples & Lindley, 2018).

In light of the recent insight into the genomic basis of salmonid migration timing (Hess et al., 2016; Micheletti et al., 2018; Narum et al., 2018; Prince et al., 2017; Thompson et al., 2019), competing theories on the evolution of the migration phenotype provide contrasting strategies to management plans (Kardos & Shafer, 2018; Oke & Hendry, 2019; Pearse, 2016; Waples & Lindley, 2018). For example, while the current framework for Chinook Salmon conservation is structured around evidence that migration timing arose through a process of parallel evolution and is therefore likely to arise again, recent studies suggest that migration timing is highly associated with a genomic region of major effect that arose from a rare mutational event (Prince et al., 2017). Further, the strong association of candidate genes greb1L/rock1 with migration timing occurs in the face of apparent gene flow of migratory types within populations (Narum et al., 2018), which raises uncertainty regarding individuals that are heterozygous for candidate markers and their respective phenotypic expression of migratory traits, frequency of occurrence, and fitness relative to homozygous individuals. Waples and Lindley (2018) point out that prior to making conservation management decisions for salmonids with respect to migration timing, it is crucial to determine the distribution of migration‐related alleles across populations and to gain a broader understanding of fitness differences underlying the migration phenotype.

In this study, we developed several markers that span 220 kb on chromosome 28 within and between candidate genes greb1L and rock1 for adult migration timing in Chinook Salmon to investigate their utility for conservation applications following questions outlined by Waples and Lindley (2018). Previous studies provided only allele frequency differences for SNP variants (Narum et al., 2018) or very few SNP markers from this candidate genomic region for individuals (Prince et al., 2017; Thompson et al., 2019). This new panel of 33 markers spanning the genomic region of major effect on chromosome 28 allowed for more thorough testing of individual phenotypic association within and among lineages of Chinook Salmon. Using individual‐level genotypes from these candidate markers, we tested for an association with migration phenotypes across three lineages that demonstrate distinct freshwater migration phenotypes but each exhibits variation for early and late arrival timing for spawning. We then tested whether the percent of phenotypic variation explained by candidate markers and the patterns of inheritance on chromosome 28 differs between each of the three lineages. Pedigree data from one of the populations enabled association tests between the candidate markers and fitness which was based on previous estimates of reproductive success (Janowitz‐Koch et al., 2019). Hereafter, we refer to alleles at candidate loci as premature or mature following previous studies in this species (Narum et al., 2018; Prince et al., 2017), but individual phenotypes as early or late arrival at spawning grounds to reflect phenotypic variation in arrival timing within each of the three lineages. It is important to point out that although a strong association has been documented between chromosome 28 and migration time, the precise point within the migration cycle that exhibits the strongest association with the genotype has not been documented. Thus, the phenotypes evaluated do not represent states of sexual maturity at freshwater entry (premature vs. mature) or timing of freshwater entry, but rather early versus late arrival timing for spawning of each lineage of Chinook Salmon.

## 2 METHODS

### 2.1 Tissue sample collection

Tissue samples were included from three distinct populations representing the three major phylogenetic lineages of Chinook Salmon in North America (coastal, interior ocean‐type, and interior stream‐type; Figure S1; Hecht et al., 20155; Narum et al., 2010; Waples et al., 2004). Adult migration phenotypes vary across these three lineages that demonstrate distinct patterns of freshwater entry but each exhibits variation for early and late arrival timing for spawning (Narum et al., 2018). Data on the precise timing of freshwater entry were not available. Therefore, samples in this study were classified by early versus late arrival for spawning within each of the three populations (i.e., lineages) and represented independent samples from those included in the original association tests (Narum et al., 2018) in order to follow best practices for validating association of candidate markers (Wray et al., 2013). Sample sizes for each collection location and phenotype category are provided in Table 1. Samples from the coastal/Lower Columbia (Cowlitz R.) and Interior ocean‐type (upper Columbia/Methow R.) lineages were classified by categorical phenotypes of early and late timing of arrival for further analyses. Both spring‐run (early) and fall‐run (late) samples were collected in 2015 from the Cowlitz River hatchery broodstock (Washington) to represent the coastal/Lower Columbia lineage (Narum et al., 2010; Waples et al., 2004). Samples chosen to represent the Interior ocean‐type lineage were collected in 2015 from fish with early arrival at Wells Hatchery (Washington; summer‐run broodstock) and fish with late arrival at Prosser Hatchery (Washington; fall‐run broodstock) that are part of a broad ranging population in the upper Columbia River demonstrating high gene flow (Moran et al., 2012; Narum et al., 2010).

**Table 1:** Sample sizes and arrival timing phenotype designations of different collection localities representing three distinct phylogenetic lineages of Chinook Salmon

Locality	Collection	Phenotype	Lineage	Region	*n*
Cowlitz	Spring, 2015	Early	Lower Columbia	WA	91
Cowlitz	Fall, 2015	Late	Lower Columbia	WA	95
Wells	Summer, 2015	Early	Interior ocean‐type	WA	95
Prosser	Fall, 2015	Late	Interior ocean‐type	WA	73
Johnson Creek	Spring/Summer, 2010–2011 & 2013–2016	Early^a^	Interior stream‐type	ID	1,593
		Late^a^			843
*Total*					2,790

^a^ Early and late arrival cutoff at ordinal day of the year = 216 (August 4th).

Samples representing the Interior Columbia River stream‐type phylogenetic lineage were collected across several years (2010–2011 and 2013–2016) from a naturally spawning population at a weir on Johnson Creek, Idaho that exclusively exhibits spring/summer entry into freshwater (Narum et al., 2018). Samples collected from the Interior stream‐type lineage provided an estimate of timing of arrival that could be treated as either a continuous trait by weir arrival date, or a categorical trait by implementing a cutoff for early versus late arrival at the weir. Finally, previous studies have determined the reproductive success of each individual passing the weir through pedigree analyses (Hess et al., 2012; Janowitz‐Koch et al., 2019), which was used for fitness components of the study (Figure S2).

### 2.2 SNP genotyping and quality control

Genotyping was completed following protocols in Janowitz‐Koch (2019) using the genotyping‐in‐thousands by sequencing method (GT‐seq; Campbell, Harmon, & Narum, 2015). DNA was extracted from fin tissue using a Chelex 100 method (Sigma‐Alrich). In addition to putatively neutral SNP markers from Janowitz‐Koch et al., (2019), 33 SNP markers were developed as candidate markers located on chromosome 28 (Table S1) that are associated with early and late arrival to spawning grounds, further referred to as premature and mature alleles, respectively (Narum et al., 2018). These 33 markers were chosen based on highly significant association results for SNPs that spanned a 220 Kb genomic region of significance on chromosome 28 that included candidate genes of greb1L, rock1, and the intergenic region between them that presumably includes the promoter (Table S1; Narum et al., 2018). To ensure adequate quality control, all samples and loci with ≥10% genotyping failure were removed from further analyses.

### 2.3 Partitioning haplotype blocks

Since candidate markers were developed in physical proximity, we first determined whether multiple SNPs on chromosome 28 were in strong linkage disequilibrium (LD). We used the program Haploview v4.2 to visualize haplotype blocks independently for each population (Barrett, Fry, Maller, & Daly, 2004; Gabriel et al., 2002). For the Lower Columbia and Interior ocean‐type populations, regions were defined as haplotype blocks (i.e., in strong LD) if the 95% D′ confidence interval was between 0.55 and 0.85. The number of pairs within a haplotype block was considered in strong LD if they were more than 0.80 times the total number of informative pairs. Upper confidence intervals that were <0.80 were considered evidence for historical recombination. These parameters were relaxed compared to default parameters and were used to ensure that potential single selective sweeps were not artificially broken into smaller haplotype blocks. For the Interior stream‐type population, haplotype blocks were estimated using the default parameters of the solid spline method in Haploview. There were zero SNPs with minor allele frequency (MAF) <0.01, and therefore, all candidate markers were included in haplotype block estimates. Haplotype block frequencies were estimated using PLINK v1.7 (Purcell et al., 2007).

### 2.4 Development of a neutral SNP marker set

To generate an unlinked (i.e., not in LD) and selectively neutral set of SNPs that could be used as a covariate for population structure and relatedness in further analyses, we used a previously published panel of 298 SNP markers and a single sex marker from the Chinook sdY region (Janowitz‐Koch et al., 2019). We removed both samples and loci with ≥10% genotyping failure. To ensure that markers from this panel were unlinked and selectively neutral, we first removed SNPs with high physical linkage using a sliding‐window approach in PLINK v1.90 (Chang et al., 2015; Purcell et al., 2007). More specifically, one SNP from a pair with an R^2^ value greater than 0.9 was removed from SNP windows of 50 shifted by five SNPs per iteration. Three candidate SNPs from chromosome 28 were also used as a positive control for the outlier analysis, since we presumed that those SNPs were not selectively neutral. We then used BayeScan V2.1 (Foll & Gaggiotti, 2008) and OutFLANK (Whitlock & Lotterhos, 2015) to determine and remove outlier SNPs. Each population was analyzed independently using program defaults, and we identified loci putatively under divergent selection with FDR <0.05. To generate a consensus list of a neutral set of markers, SNP markers identified as outliers in any of the three populations were removed from further analysis. The R package adegenet (Jombart, 2008) was used to create a principal component analysis (PCA) for candidate chromosome 28 markers and putatively neutral markers to contrast patterns of adaptive versus neutral structure.

### 2.5 Genome‐wide association analyses

We used GWAS to confirm an association between markers on chromosome 28 and early versus late timing of arrival within each population. First, we ran a GWAS using the full panel of 33 SNP markers on chromosome 28. There were zero SNPs with MAF < 0.01, and therefore, all candidate markers were included in association analyses. The GWAS was conducted using the mixed linear model (MLM) function implemented in the GAPIT R package, which allows for the inclusion of fixed and random effects to account for population structure and relatedness, respectively (Lipka et al., 2012; Zhang et al., 2010). For the Lower Columbia and Interior ocean‐type populations, arrival date was binary and represented numerically as one or two for early versus late arrival timing, respectively. For the Interior stream‐type population, ordinal date of arrival was used as a continuous variable in the models. However, the distance from the ordinal date of arrival to the median arrival day of 216 was used to bin data to allow for comparisons across lineages. To account for variation in arrival timing across years in the Interior stream‐type population, the year of return (i.e., arrival) was included as a fixed effect in the model. Genetic sex was also included as a fixed effect for all models. If genetic sex was not available from the sex marker, phenotypic sex was used instead (<5% of individuals). In the program GAPIT, the first three PCs to account for neutral population structure were included as a fixed effect for each association test. The neutral markers were then used to generate a kinship matrix using the VanRaden method (VanRaden, 2008), which was included as a random effect in the models. Parameter estimates from univariate associations between each SNP and arrival timing in R version 3.5.3 were used to calculate the percentage of premature‐versus mature‐associated alleles for each individual (RCore, 2016).

Due to the fact that our markers were in high LD, we used the program BLINK (Bayesian‐information and Linkage‐disequilibrium Iteratively Nested Keyway) as implemented in the GAPIT R package to test the effect of multiple markers simultaneously (Huang, Liu, Zhou, Summers, & Zhang, 2018). BLINK uses a stepwise mixed‐model regression with forward inclusion and backward elimination to perform model optimization while accounting for SNPs in high LD (Pearson correlation coefficient = 0.999). By using this approach, the proportion of phenotypic variance explained by the most causal SNPs was maximized. If multiple SNPs were in high LD with redundant explanatory power, the first SNP from a block was chosen. All fixed and random effects, including those accounting for population structure and relatedness, remained consistent between MLM and BLINK analyses.

To determine whether there was an association between fitness and candidate markers on chromosome 28, we used a subset of data that were available from Johnson Creek that included reproductive success (i.e., number of returning adult offspring for each spawning adult; *n* = 1,346; see Janowitz‐Koch et al., 2019) as a continuous variable in GAPIT. We first ran an MLM using the full panel of 33 SNP markers on chromosome 28 to test for an association with fitness. Year of return, sex, age, and population structure (based on the neutral marker list) were included as fixed effects in the model, and kinship from neutral markers was included as a random effect. Analyses were then run separately for males and females, and sex was removed as a fixed effect from the models. Finally, we ran a stepwise analysis using the BLINK model described above to account for SNPs in high LD. Because the number of offspring demonstrated a negative binomial distribution (see Figure S2) and GWAS in GAPIT assumes a normal distribution, we ran an independent association for males and females separately to estimate the effects of year, age, arrival time to spawning grounds (analyzed using linear and quadratic second‐order orthogonal polynomial contrasts), and any SNPs that significantly predicted arrival timing in BLINK models. By using SNPs that significantly predicted arrival timing, we were able to determine the relationship between chromosome 28 markers, fitness, and arrival timing. Using a negative binomial distribution model and a log link function, we used the stepwise AIC regression procedure to determine the best fit model. To ensure that all models were fit to the same number of observations, individuals with missing genotypes were removed from the analysis (*n* = 75 out of 1,346; 6%).

The stepwise negative binomial GLMs were run using the glm.nb and stepAIC function as part of the MASS package in R (Venables & Ripley, 2013). We report both raw and adjusted P‐values for all models using the Benjamini–Hochberg FDR‐controlling procedure (Benjamini & Hochberg, 1995). The phenotypic variation explained by the MLMs with and without the strongest associated SNP was assessed with a likelihood‐ratio‐based statistic denoted R^2^ (Sun et al., 2010). To determine R^2^ values of each SNP for BLINK models, we used a custom R script to estimate the difference between R^2^ values with each SNP and without each SNP (available on Dryad). It is important to point out, however, that there are limitations of using R^2^ values to explain the percent of variation in models with binary response variables (i.e., early vs. late). Therefore, we also include results from a genomic relatedness‐matrix restricted maximum likelihood (GREML) method implemented in the genome‐wide complex trait analysis (GCTA) software to estimate the variance in arrival timing explained by genetic variance (VG/VP; Yang et al., 2010; Yang, Lee, Goddard, & Visscher, 2011; Yang, Zeng, Goddard, Wray, & Visscher, 2017). This method uses relatedness as a random effect in a mixed linear model to predict phenotypic relatedness through restricted maximum likelihood. We estimated the variance in arrival timing from variance of the significant SNPs determined by BLINK on chromosome 28. The first three PCs from MLM in GAPIT were included as a fixed effect to account for neutral population structure, and sex was also included as a fixed effect. For both the Lower Columbia and Interior ocean‐type population, arrival timing was considered a bivariate phenotype. To ensure consistency between all analyses, we estimated VG/VP in a subpopulation of nonfounders (*n* = 1642) for the Interior stream‐type population and also added year as a fixed effect. Power calculations using the GCTA‐GREML power calculator indicated <10% power to estimate VG/VP with high accuracy (Visscher et al., 2014). Therefore, results from the GREML analysis should be interpreted with caution.

## 3 RESULTS

### 3.1 Data trimming and development of a neutral marker set

After removing samples that genotyped at ≤90% of markers, a total of 2,790 individuals were used for further analyses (Table 1). After trimming neutral markers with <90% coverage, 292 out of 298 loci remained in the dataset. We then used an LD and MAF‐trimmed dataset of 196 SNPs in an outlier test. Each population was analyzed independently with the same set of 196 SNPs. After trimming loci that were non‐neutral based on OutFLANK and BayeScan results (*n* = 10; Table S2) in addition to one marker on chromosome 28 that was not deemed an outlier, 185 SNPs remained in the final neutral marker list (Table S3).

### 3.2 PCA clustering of neutral and candidate markers

**Figure 1:**
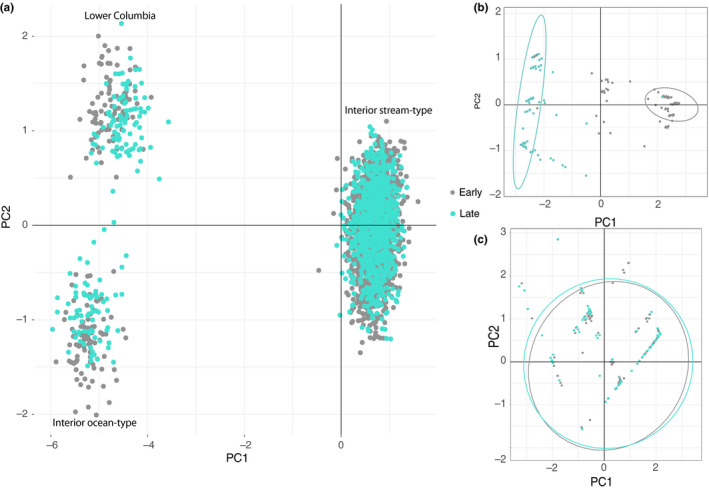
PCA of genetic variation in Chinook Salmon for (a) 185 neutral SNP markers, (b) 33 chromosome 28 markers for the Lower Columbia and Interior ocean‐type populations combined, and (c) 33 chromosome 28 markers for the Interior stream‐type population. Ellipses represent 95% confidence levels

A series of PCA results demonstrated clear differences in clustering between putatively neutral markers and those associated with timing of arrival to spawning grounds in two of the three lineages (Figure 1). A PCA with neutral markers showed three distinct clusters of individuals that grouped by population, with no relationship to phenotype (early vs. late arriving fish were distributed proportionately throughout each population; Figure 1a). In contrast, a PCA of the 33 markers on chromosome 28 showed that the variation in candidate markers demonstrated a clear pattern of separation between early and late arrival timing in the Lower Columbia and Interior ocean‐type populations (Figure 1b). The pattern was investigated separately for the larger set of individual samples from the Interior stream‐type lineage that had continuous data for arrival timing including those fish that were intermediate between early and late arrival timing peaks. There was substantial overlap between early and late arriving samples for the Interior stream‐type lineage (Figure 1c), demonstrating less distinction by arrival timing compared to the Lower Columbia and Interior ocean‐type populations. When samples were separated by year of return, the extent of overlap between early and late samples differed by sample year (Figure S3).

### 3.3 Haplotype block estimation

**Figure 2:**
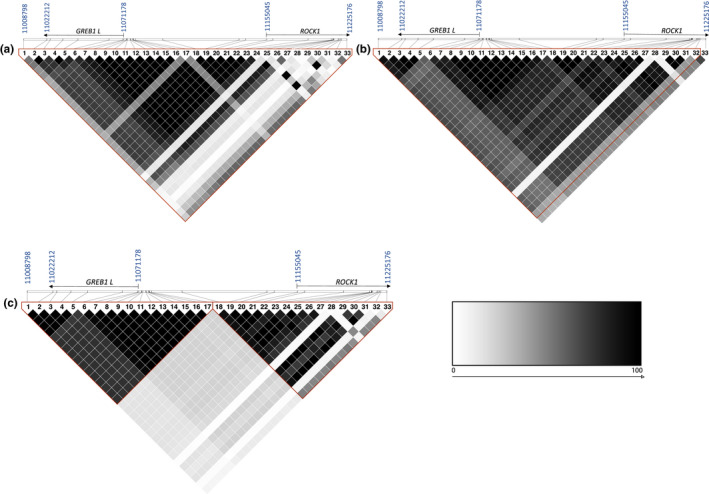
Estimated haplotype blocks in three lineages of Chinook Salmon for the (a) Lower Columbia population, (b) Interior ocean‐type population, and (c) Interior stream‐type population. Blocks span a region on Chinook chromosome 28 starting with 11.001 Mb and ending with 11.225 Mb, which encompasses candidate genes greb1L, rock1, and the intergenic region. SNP number on the x‐axis corresponds to the markers identified in Table S1, while the genome position of markers on chromosome 28 is depicted in the gene diagrams above the x‐axis. Start and stop locations represented in blue text; haplotype blocks represented by red lines. Values within each block are R^2^ values and represent a gray scale continuum from R^2^ = 0 (white boxes) through R^2^ = 100 (black boxes)

We then used Haploview to visualize potential haplotype blocks. For both the Lower Columbia and the Interior ocean‐type populations, one haplotype block with 32 SNPs was estimated (31 out of 32 SNPs were the same between the two populations; Figure 2a, b; Table S4). For the Interior stream‐type population, two haplotype blocks were estimated (one block with 17 SNPs and another block with 16 SNPs; Figure 2c; Table S4) and there was a strong break in LD between markers 17 and 18 that was not apparent in populations from the other two lineages. Although there were zero SNPs with minor allele frequency (MAF) <0.01, it is worth noting that SNP number 28 (Ots28_11202863) displayed a lower MAF (Lower Columbia = 0.046, Interior ocean‐type = 0.066, and Interior stream‐type = 0.014) than other candidate markers (range of MAF was 0.014–0.464 for all lineages).

### 3.4 Association analyses

**Figure 3:**
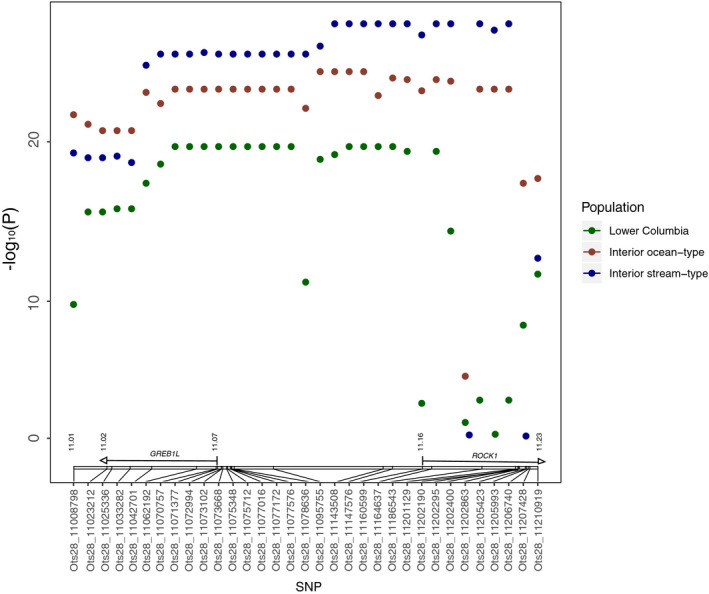
Association of each candidate SNP marker with arrival timing within three lineages of Chinook Salmon from MLM as implemented in GAPIT. The genome position of markers on chromosome 28 (NCBI accession GCA_002831465.1) is depicted in the gene diagrams above the x‐axis. Y‐axis represents − log_10_ (FDR‐corrected *p*‐value)

Initial association tests in GAPIT using MLM with all 33 candidate markers validated that the majority of markers were significantly associated with arrival timing for all three lineages (FDR‐corrected *p* < .05; Figure 3; Table S5). The strongest association was consistently observed for markers in the intergenic region upstream of both greb1L and rock1, or within the rock1 gene. Markers located in 3’ UTR regions or downstream of each gene displayed lower signals of association (Figure 3; Table S5).

We then used a stepwise approach as implemented in BLINK to determine the most significant SNPs after accounting for SNPs in high LD. For the Lower Columbia population, the marker Ots28_11071377 was significantly associated with arrival timing (FDR‐corrected *p* < .05; Table 2) and for the Interior ocean‐type population, the marker Ots28_11143508 was significantly associated with arrival timing. Two markers (Ots28_11078636 and Ots28_11202190) were significantly associated with arrival timing for the Interior stream‐type population, one from each haplotype block (FDR‐corrected *p* < .05; Table 2). Across all populations, R^2^ values provided evidence that a large amount of phenotypic variation was explained by these significant SNPs in each lineage, with 28.7% for the Lower Columbia population, 77.9% for the Interior ocean‐type population, and 4.7% for the Interior stream‐type population (Table 2) once removing the proportion of variation explained by neutral markers. Using the GREML method implemented in GCTA, we found that VG/VP = 69.8%, 98.1%, and 3.6% for the Lower Columbia, Interior ocean‐type, and Interior stream‐type populations, respectively.

**Table 2:** Significant SNPs based on GWAS results from BLINK for candidate markers on chromosome 28 predicting arrival timing for three lineages of Chinook Salmon

	SNP	Gene	P‐value	FDR‐adjusted P‐value	R^2^ of model without SNP	R^2^ of model with SNP	R^2^ of SNP
Lower Columbia	Ots28_11071377	intergenic	3.59E−51*	1.19E−49*	0.567	0.854	0.287
Interior ocean‐type	Ots28_11143508	intergenic	4.76E−31*	1.57E−29*	0.212	0.991	0.779
Interior stream‐type	Ots28_11078636	intergenic	4.81E−14*	1.59E−12*	0.052	0.099	0.047
	Ots28_11202190	rock1	0.000891*	0.014708*	0.052	0.099	0.047

Notes

FDR represents Benjamini–Hochberg‐corrected *p*‐values; *p < .05.

**Figure 4:**
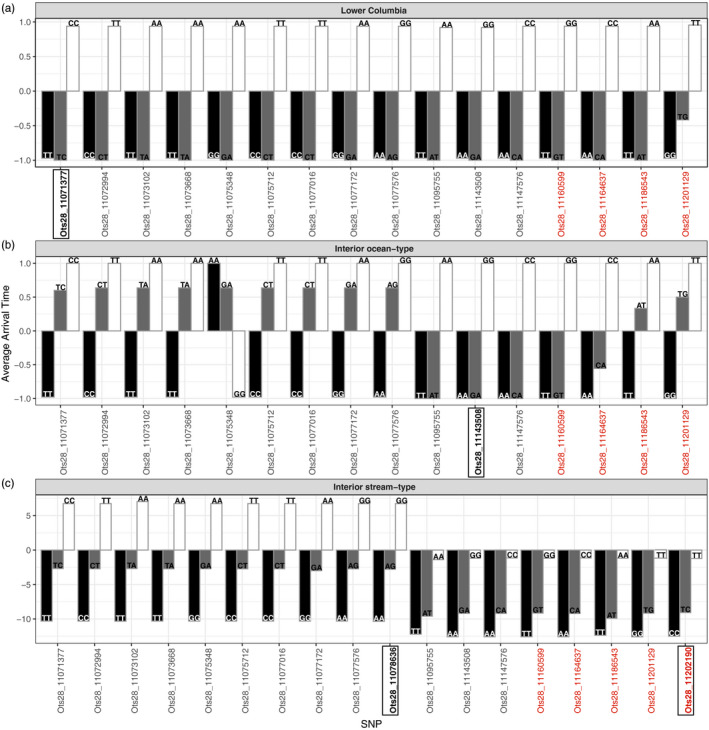
Top significant SNPs from MLM as implemented in GAPIT (see Figure 3) and relationship to the average arrival timing phenotypes in Chinook Salmon. Results are presented separately for each locus for each population. Black and white bars represent homozygous premature and mature genotypes, respectively, and gray bars represent the heterozygous genotypes. Y‐axis represents early (−1) and late (1) binary phenotypes for the Lower Columbia (a) and Interior ocean‐type (b) populations. For the Interior stream‐type population (c), y‐axis represents the distance from ordinal day 216 (August 4th), the cutoff used for early versus late arrival to spawning grounds. Significant SNPs from BLINK results are represented in bold boxes. Black SNPs represent those within the intergenic region, while red SNPs represent those located on rock1

Genotypic classes of the top significant SNPs in GAPIT’s MLM (Figure 3) showed a strong relationship with average arrival timing across all three lineages (Figure 4). In the Lower Columbia lineage (Figure 4a), fish that were homozygous for the premature allele were early arriving fish that arrive in the spring in contrast to those with the alternative homozygous genotype for the mature allele that were arriving to the spawning grounds later (in fall). Fish that were heterozygous for the premature allele were strongly skewed toward early arrival. Similarly, in the Interior ocean‐type lineage (Figure 4b), fish that were homozygous for the premature allele were typically early arriving fish that arrive in summer and homozygotes for the mature allele were generally late arriving fall fish. This pattern was even more evident in or near the top significant SNP. Fish that were heterozygous for the premature allele were also strongly skewed toward early arrival, which again became more evident near the top significant SNP in rock1, suggesting a pattern of dominant inheritance for the premature allele in rock1. In the Interior stream‐type lineage (Figure 4c), arrival timing was a continuous variable rather than categorical as in the other two lineages, so the y‐axis was scaled to number of days before or after the median arrival day 216 (shown as zero on the y‐axis in Figure 4c). Interior stream‐type demonstrated a pattern where fish with homozygous genotypes for premature alleles were earlier arriving fish, while homozygous fish for the mature allele arrived later on average. Heterozygous fish were intermediate in arrival timing but were once again skewed toward early arrival timing especially for markers in or near rock1, closest to the top two significant SNPs (Figure 4c).

**Figure 5:**
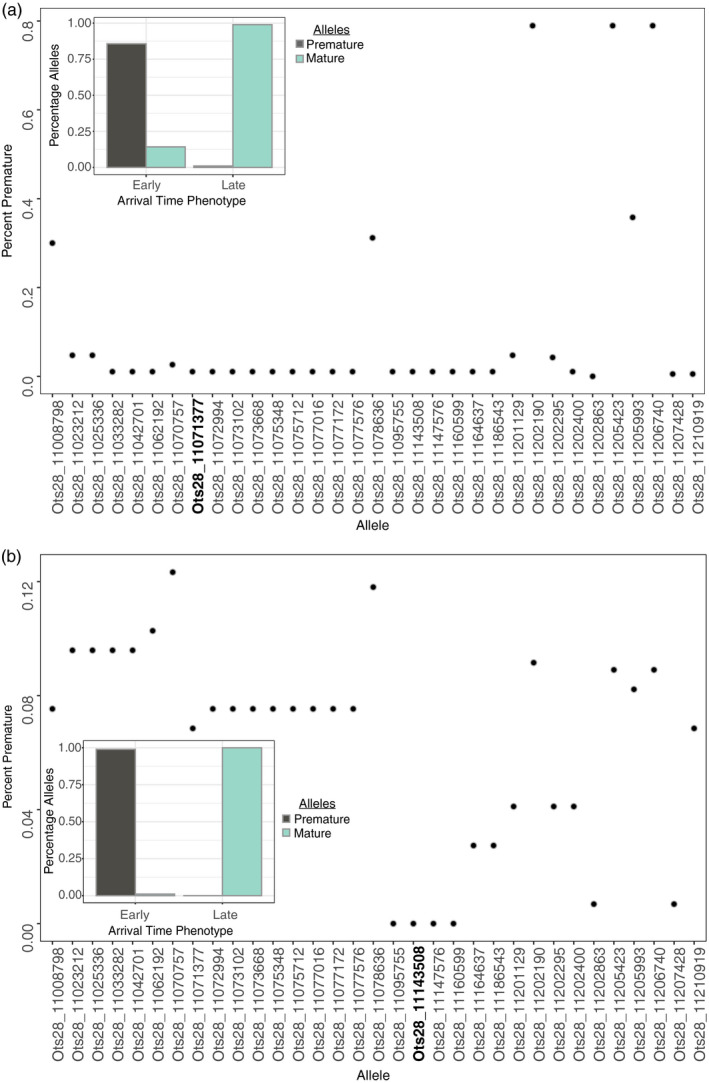
Percentage of premature alleles for the top significant SNPs from MLM as implemented in GAPIT in late arriving fish in the Lower Columbia (a) and Interior ocean‐type (b) populations. Bar graph inset represents the top (bolded) significant SNP from BLINK results with gray and turquoise bars representing premature and mature alleles, respectively, while x‐axis represents the phenotype designation of early versus late arrival timing

Overall, the percentage of premature alleles in late arriving fish varied by population (Figure 5). For the Lower Columbia population, the percentage of premature alleles in the late arriving fish remained close to zero at or near the top significant SNP (Figure 5a). When examining the top SNP more closely, there remained a consistent relationship where early arriving fish had a relatively large percent of premature compared to mature alleles, while late arriving fish had almost entirely all mature alleles (Figure 5a; Figure S4). For the Interior ocean‐type population, the pattern appeared similar to the Lower Columbia population, with the percentage of premature alleles at or near zero in the region encompassing the most significant SNP. Overall, at the most significant SNP, early arriving fish had close to 99% premature alleles, while late arriving fish had 100% mature alleles (Figure 5b; Figure S5). It was noteworthy that less significant markers in each lineage demonstrated inconsistent patterns of the percentage of premature alleles, ranging from 0% to 80% (Figure 5).

**Figure 6:**
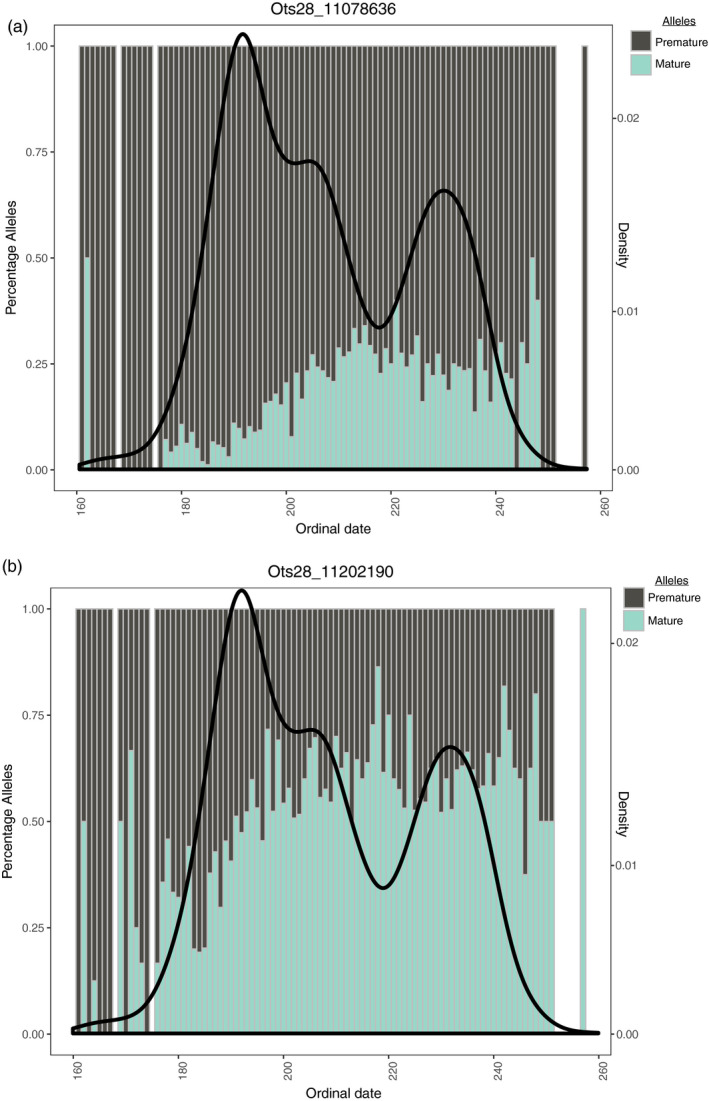
Percentage of premature and mature alleles for the top two significant SNPs (a and b) from BLINK results in early and late arriving fish in the Interior stream‐type population. Gray and turquoise bars represent premature and mature alleles, respectively, while x‐axis represents ordinal date. Density plot of ordinal date is represented by the black line on the secondary y‐axis. All return years are combined

Results were less consistent for the Interior stream‐type populations, but early arriving fish typically demonstrated a higher percentage of premature alleles (Figure 6) for the top two significant SNPs relative to later arriving fish. However, late arriving fish also retained a relatively high percentage of premature alleles (Figure 6), particularly for the significant SNP from the first linkage block (Ots28_11078636; Figure 6a). Upon a closer examination of the top two significant SNPs broken out by each of six return years (2010–2011, 2013–2016; Figure S6), fish alternated between having a majority of premature or mature alleles depending on the year.

**Figure 7:**
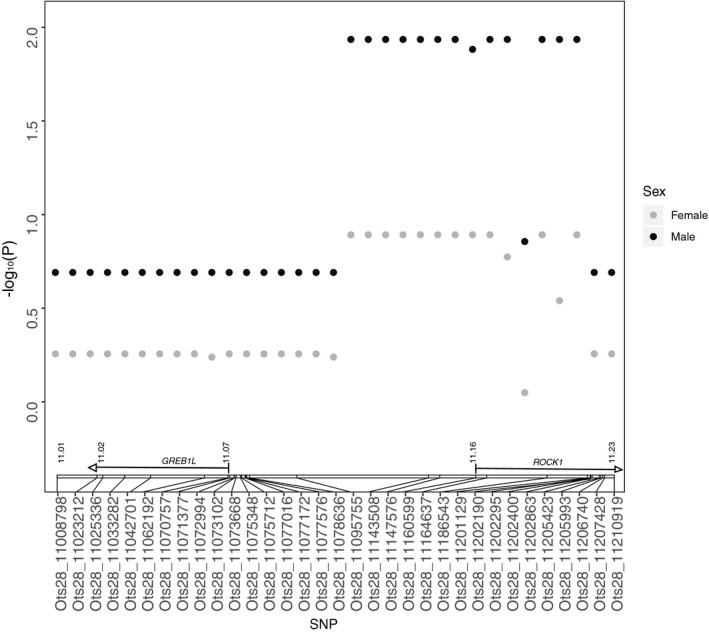
Association of each candidate SNP marker with fitness within the Interior stream‐type population from MLM as implemented in GAPIT. The genome position of markers on chromosome 28 (NCBI accession GCA_002831465.1) is depicted in the gene diagrams above the x‐axis. Y‐axis represents − log_10_ (FDR‐corrected *p*‐value). Sexes are presented separately

In the Interior stream‐type population, previous results from pedigree analyses provided fitness for each spawning adult in 2010 (*n* = 824) and 2011 (*n* = 522; Janowitz‐Koch et al., 2019). Therefore, an additional GWAS was completed in GAPIT to determine whether chromosome 28 markers were significantly associated with fitness. For the MLM association test in GAPIT using all 33 candidate SNPs combined across sexes, 13 SNPs were significantly associated with fitness (FDR‐corrected *p* < .05; Table S6; Figure 7). When sexes were analyzed separately, there were no SNPs significantly associated with fitness for females (FDR‐corrected *p* > .05; Table S6), but 13 SNPs were significantly associated with fitness for males (FDR‐corrected *p* < .05; Table S6). Overall, the only significant markers were within or upstream of the rock1 gene or upstream of greb1L, but none were in greb1L (Table S6; Figure 7). When using the BLINK stepwise approach to determine the most significant SNPs after accounting for SNPs in high LD, zero SNPs were significant for males, females, or sexes combined (Table S6).

**Figure 8:**
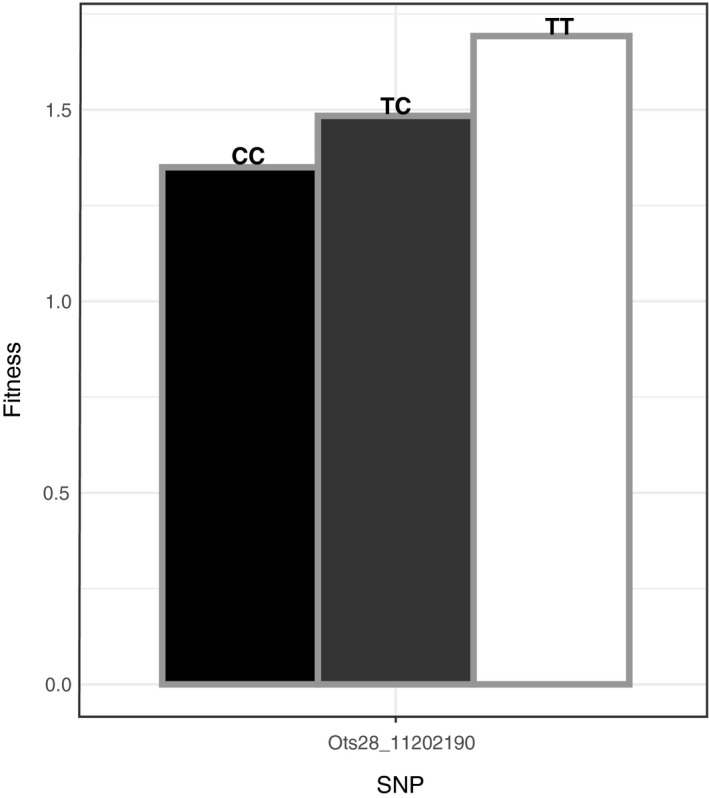
Top significant SNP from BLINK predicting arrival timing as implemented in GAPIT. Data shown are the relationship between one of the two SNPs predicting arrival timing that also demonstrated a significant relationship to the fitness phenotype in the Interior stream‐type population of Chinook Salmon combined across sexes. Black and white bars represent homozygous premature and mature genotypes, respectively, and gray bars represent the heterozygous genotype. Fitness on the y‐axis reflects average reproductive success for parents of each genotypic class based on previous pedigree studies (Janowitz‐Koch et al., 2019)

To expand upon the potential effect of various factors on individual fitness in the Interior stream‐type population, we used a negative binomial distribution model and a log link function to examine factors including timing of arrival to spawning grounds, sex, year, and age in addition to genotypic data for the two candidate markers that significantly predicted arrival time in this population (Ots28_11078636 and Ots28_11202190). Models were run separately for each sex and with sexes combined. The best fit model for sexes combined, males, and females included predictors of quadratic arrival time, sex, year, age, and one (out of two) of the candidate markers on chromosome 28 (Table 3). When sexes were combined, we found that arrival time, sex, year, age, and marker Ots28_11202190 significantly predicted fitness (FDR‐corrected *p* < .05; Table 3; Figure 8). For a model that included males only, we found that arrival time, year, age, and marker Ots28_11202190 significantly predicted fitness (FDR‐corrected *p* < .05; Table 3; Figure S7). However, the significant quadratic term in the model suggests that the relationship with arrival timing and fitness is nonlinear. When testing a female‐only model, we found that year was the only significant predictor of fitness (FDR‐corrected *p* < .05; Table 3; Figure S7). Overall with sexes combined, Ots28_11202190 demonstrated a clear pattern with fitness of fish that were homozygous for the mature allele having the highest average fitness, heterozygous individuals showing an intermediate fitness phenotype, and homozygous fish with the early allele having the lowest average fitness (Figure 8).

**Table 3:** Final models predicting fitness under a negative binomial distribution for the Interior stream‐type population

Final Model	Sexes combined				Males				Females			
	Fitness = Arrival Time + (Arrival Time)^2^ + Sex +Year + Age +Ots28_11202190				Fitness = Arrival Time + (Arrival Time)^2^ + Year +Age + Ots28_11202190				Fitness = Arrival Time + (Arrival Time)^2^ + Year +Age + Ots28_11202190			
Model Results	Estimate	*SE*	Raw *p*‐value	FDR‐adjusted *p*‐value	Estimate	*SE*	Raw *p*‐value	FDR‐adjusted *p*‐value	Estimate	*SE*	Raw *p*‐value	FDR‐adjusted *p*‐value
Arrival Time	1.896e−02	7.657e−02	.804398	.852601	2.854e−01	1.103e−01	.00966*	.0147867*	−0.2369670	0.1107510	.032384*	.0607058
(Arrival Time)^2^	−6.011e−05	1.784e−04	.736226	.852601	−6.919e−04	2.581e−04	.00736*	.01472*	0.0005430	0.0002571	.034689*	.0607058
Sex (Male)	−2.616e−01	7.339e−02	.000364*	.000819*	NA	NA	NA	NA	NA	NA	NA	NA
Year (2011)	−6.984e−01	9.516e−02	2.14e−13*	6.42e−13*	−7.990e−01	1.231e−01	8.67e−11*	3.468e−10*	−0.5373019	0.1488600	0.000307*	.002149*
Age (4)	9.312e−01	1.149e−01	5.22e−16*	4.698e−15*	9.853e−01	1.228e−01	1.05e−15*	8.4e−15*	NA	NA	NA	NA
Age (5)	1.289e + 00	1.717e−01	5.90e−14*	2.655e−13*	1.426e + 00	4.452e−01	.00136*	.00362667*	0.3086492	0.1673365	.065113	.07596517
Ots28_11202190 (TT)	2.786e−01	9.906e−02	.004918*	.0088524*	2.878e−01	1.404e−01	.04040*	.04617143*	0.2560113	0.1381542	.063870	.07596517
Ots28_11202190 (TC)	1.022e−01	8.941e−02	.253036	.379554	1.641e−01	1.251e−01	.18960	.1896	0.0367089	0.1264652	.771611	.771611

Notes

Results presented are estimated model coefficients for fitness (i.e., number of offspring) as a function of quadratic arrival time, sex, year, age, and two markers on chromosome 28. Coefficients shown are from the best fitting generalized linear model with a negative binomial distribution and a log link function and include estimated standard errors (SE) and p‐values (both raw and Benjamini–Hochberg FDR‐corrected); *p < .05. Results are presented separately for sexes combined, males only, and females only.

## 4 DISCUSSION

Association tests confirmed that candidate markers on or near greb1L and rock1 on chromosome 28 were strongly associated with timing of arrival for spawning in populations representing three distinct lineages of Chinook Salmon (Lower Columbia, Interior ocean‐type, and Interior stream‐type), supporting previous studies identifying this candidate region of the genome (Hess et al., 2016; Micheletti et al., 2018; Narum et al., 2018; Prince et al., 2017; Thompson et al., 2019). Overall, results indicated that a high percentage of phenotypic variation for arrival to spawning grounds was explained by candidate markers on chromosome 28 for two of the populations representing the Lower Columbia and Interior ocean‐type lineages. However, the percentage of phenotypic variation for arrival timing that was explained by the top two markers in the Interior stream‐type population was much lower, potentially reflecting polygenicity, stronger environmental effects, or differences in trait designations in this population. These results were similar to a previous study that demonstrated a significant but weaker association of migration timing for spawning in the Interior stream‐type population relative to the other two lineages (Narum et al., 2018).

For Lower Columbia and Interior ocean‐type lineages of Chinook Salmon, early arriving fish demonstrated a much greater proportion of premature alleles across markers than late arriving fish. The proportion of premature alleles rose to nearly 100% in or near the most significant markers, suggesting that the markers that accounted for the most variation in arrival time potentially provide the most accurate representation of allele frequencies for this trait. We observed the same pattern for late arriving fish that had a higher percentage of mature alleles, with almost 100% in or near the top significant markers. For both populations, the percent of premature alleles in the late arriving populations was close to zero at or near the top significant SNPs. This suggests that while standing genetic variation for early arrival may exist at very low levels within late arriving fish, it is unlikely to prevent extirpation under a case of complete loss of the early migrating phenotype, a finding that has been seen in other studies (Thompson et al., 2019).

For the Interior stream‐type population, there were still a relatively high percentage of premature alleles found within late arriving fish and mature alleles found within early arriving fish. These results suggest that genetic variation for both early and late arrival to spawning grounds may exist within some interior populations. However, it is possible that the cutoff used to define binary early versus late arrival phenotypes in this population does not adequately capture true variation in maturation timing. Unlike the other populations examined that have variable freshwater entry timing, fish from the Interior stream‐type lineage only enter freshwater as sexually premature but may exhibit a bimodal pulse in the final ascent to the spawning grounds. Thus, Interior stream‐type fish do not experience the same trade‐off as the other two populations examined in this study whereby fish maturing in the ocean experience potentially longer access to feeding but a higher predation risk (Quinn et al., 2016). Therefore, the early versus late distinction in the Interior stream‐type population is based on arrival behavior (i.e., final ascent to the spawning grounds), which may not be equivalent to premature versus mature freshwater entry patterns. A recent study (Thompson et al., 2019) suggests that genotypes in the greb1L region of chromosome 28 may be better predictors of general adult migration for freshwater entry than upriver passage (arrival timing for spawning), but precise phenotypes for freshwater entry and spawning dates are needed to investigate further. Thus, future studies aimed at determining the relationship between timing of freshwater entry, arrival for spawning, and sexual maturation would provide more direct analyses of genotype to phenotype relationships in Chinook Salmon across broad geographic regions.

The current study also revealed strong patterns of LD across the 220 Kb region on chromosome 28. However, there was a distinct break in the intergenic region for the Interior stream‐type population that was not observed in the other two populations examined and a PCA of chromosome 28 markers also confirmed that the early versus late arrival phenotype does not cluster independently for the Interior stream‐type population compared to the Lower Columbia and Interior ocean‐type populations. These results were reflected in differences in the distribution of alleles across arrival timing for different years in the Interior stream‐type population. Taken together, this suggests that while there is a significant association between candidate markers and the arrival phenotype in the Interior stream‐type population, yearly environmental variation could play a role in both phenotypic expression of the trait in the form of plasticity (intra‐generational) and in selection for the trait in certain years compared to others (inter‐generational). Previous research has shown that ocean and stream conditions directly affect migration timing (Anderson & Beer, 2009; Hodgson, Quinn, Hilborn, Francis, & Rogers, 2006; Jonsson, Jonsson, & Hansen, 2007; Mundy & Evenson, 2011) and can drive selection on the trait (Crozier, Scheuerell, & Zabel, 2011; Kovach, Gharrett, & Tallmon, 2012; Quinn, Hodgson, Flynn, Hilborn, & Rogers, 2007). Thus, it remains likely that variation in arrival time to spawning grounds in the Interior stream‐type population is influenced by annual environmental conditions that could interact with genotypes at greb1L and rock1.

Across all three populations, there was evidence that heterozygotes at the most significant candidate markers demonstrated early arrival timing which provides information regarding the mode of inheritance patterns beyond previous studies (Prince et al., 2017; Thompson et al., 2019). Specifically, for SNPs in or near the top significant markers, particularly markers in the rock1 gene, heterozygous individuals were highly skewed toward early arrival for spawning, suggesting dominant inheritance for premature alleles in rock1, and to a lesser extent, the intergenic region between greb1L and rock1. These results suggest that the alleles for early arrival are not masked (recessive) in the heterozygous state and may be dominant in rock1, and thus would be readily lost from populations under natural selection against early arrival (Thompson et al., 2019). However, in the Interior stream‐type population, the relationship between alleles and arrival timing to spawning grounds was dependent on year, providing additional support that balancing selection could be driving the maintenance of the alternative arrival time phenotypes. We also show that individuals heterozygous for the two chromosome 28 markers that significantly predicted arrival timing demonstrated a trend toward intermediate fitness in the Interior stream‐type population. Therefore, in scenarios of strong selection favoring late arrival, the premature migration alleles might not be maintained through heterozygous individuals in this population since these fish would potentially demonstrate earlier arrival timing and lower fitness relative to late arriving individuals. However, this result was largely driven by males in the dataset, with the fitness difference only being significant between the premature versus mature homozygous genotypes (but not the heterozygous genotype). Future studies aimed at modeling inheritance patterns across lineages may determine whether the dominance inheritance patterns observed are significant for various phenotypes related to arrival timing.

While balancing selection may maintain phenotypic variation for both early and late migration phenotypes through disruptive selection related to high stream temperatures in the middle of migration (Narum et al., 2018), other scenarios for balancing selection may also include sexual selection and negative frequency‐dependent selection. In this study, fitness was significantly associated with one marker in particular in males, but not females, that was located within rock1 (Ots28_11202190). Although not measured in this study, these results could be indicative of intralocus sexual conflict whereby distinct optimums in migration timing differentially impact fitness for each sex, maintaining standing genetic variation at loci association with migration time (Cox & Calsbeek, 2009). Previous work in salmonids has demonstrated evidence of differential selection on migration time for males and females (Dickerson, Brinck, Willson, Bentzen, & Quinn, 2005; Janowitz‐Koch et al., 2019; Kodama, Hard, & Naish, 2012; McLean, Seamons, Dauer, Bentzen, & Quinn, 2007). However, it is important to point out that while we found markers that significantly predicted fitness for males and combined sexes using a model that did not account for LD, we did not find any significant markers using a stepwise approach after accounting for LD. Using a third analysis that accounted for non‐normality of fitness data, we again found that marker Ots28_11202190 significantly predicted fitness for males and combined sexes, but not for females. Therefore, the GWAS results for fitness should be interpreted with caution and may only apply to this single population, but future studies should expand on the relationship between arrival time and fitness, along with the possibility of intralocus sexual conflict, in other populations such as the Lower Columbia and Interior ocean‐types.

As suggested by Waples and Lindley (2018), gaining a broader representation for genetic variation in migration timing across populations, examining patterns of inheritance in rock1/greb1L, and determining phenotypes of heterozygotes are all areas of research that require careful evaluation in individual populations of Chinook Salmon prior to making conservation decisions. The results of this study provide strong support that timing of arrival is driven by genetic variation at adjacent candidate genes greb1L and rock1 on chromosome 28 across populations representing distinct lineages of Chinook Salmon. After accounting for LD, the most significant SNPs were located within rock1 or upstream of rock1 and greb1L, providing more precise information regarding the specific candidate genes underlying phenotypic variation for alternative arrival time phenotypes in Chinook Salmon. We also show that when examining highly significant SNPs predicting arrival time, premature alleles were present at very low frequency in late arriving populations from the Lower Columbia and Interior ocean‐type lineages, which provides limited or no potential for recovery of early fish from populations that are nearly fixed for mature alleles (Waples & Lindley, 2018). Furthermore, alleles for early arrival follow patterns suggestive of dominance inheritance in rock1 and therefore readily lost from populations under natural selection against the early phenotype. However, populations from the Interior stream‐type lineage may have standing genetic variation that selection could act upon under scenarios of differing environmental conditions, which would be dependent on the amount of gene flow between populations, inheritance pattern of premature alleles, and strength of selection. By using a systematic approach in this study to examine an important phenological trait in salmonids, we broadened our understanding for genetic variation and adaptive potential of Chinook Salmon populations, providing additional information to inform conservation management decisions.

## CONFLICT OF INTEREST

None declared.

## Supporting information

Supporting information

## Data Availability

Phenotype data, genotype data, and custom R scripts are available on the Dryad Digital Repository: https://doi.org/10.5061/dryad.f1vhhmgtc
