# Supplementary material for: Validation and association of candidate markers for adult migration timing and fitness in Chinook Salmon
Source: Evol Appl. 2020 Jun 8;13(9):2316–32. doi: 10.1111/eva.13026 (PMC7513726; doi:10.1111/eva.13026)
Supplement: Supplementary file 1 — Supporting information [file EVA-13-2316-s001.docx]

**Table S1.** List of 33 SNPs located on chromosome 28 that were developed as candidate markers associated with early and late arrival timing to spawning grounds in Chinook Salmon. The position of the target SNP on chromosome 28 is reflected in the name of the marker following the underscore based on genome assembly in NCBI (accession GCA_002831465.1; Narum et al. 2018). Bold and underlined markers in the last column represent alleles that were significantly associated with the early, compared to the late, arrival phenotype in all three lineages with the exception of Ots28_11202863, Ots28_11205993, and Ots28_11207428 which were not significant in one of the three populations. Premature-associated alleles for Ots28_11205993 are not represented because the relationship to the arrival time phenotype was not consistent across all populations.

| **Number** | **SNP** | **Gene** | **Forward Primer** | **Reverse Primer** | **Probe** |
| --- | --- | --- | --- | --- | --- |
| 1 | Ots28_11008798 | abhd3 | TAACACTGGACCATAGAGCAGAAG | TCTATGAATCTATGCCAAGTTCCA | CCTGT[**T**/C]TTCACCAAGC |
| 2 | Ots28_11023212 | greb1L | AGAAAGCCATCATCATGAGACC | ACAAACAAACAAAAATGGTCAGAA | AACGTGACAC[**A**/G]AT |
| 3 | Ots28_11025336 | greb1L | TGCAATATAGAACAAATCCGAAAA | AATAACCCTTGGCTTCACATACAT | CAATGAAGTT[**A**/C]ATTTAATTGG |
| 4 | Ots28_11033282 | greb1L | GGCTTTCTGATGATCTTGAACTTT | AGTGTGAGAGAGAGGAAGTCCCTA | TAAAAATG[**G**/A]TTGATATGTA |
| 5 | Ots28_11042701 | greb1L | CATTTGGTAACAGTCTTTGCACTT | CTGGAGTCTTTGACCATTTTTAGG | CGGTCTAT[**A**/G]ACTTGGGTGG |
| 6 | Ots28_11062192 | greb1L | AGATGATATGGATTTGCTGTGTGT | TTGAACATAACGATCAGAGAAAGA | TTCTCAAGTC[**C**/G]TACTCAACTG |
| 7 | Ots28_11070757 | greb1L | TTTTGGAACCCTTTTTACTACGAG | ACATCAGTATAGCAGAGGAGAGGG | ACCCATGAAT[**A**/G]AGGACGAGAG |
| 8 | Ots28_11071377 | intergenic | ATTTGCTGTGTGTGGAGTGAAT | GTAGTGACAGATGCTCTTGGAGG | CATCTTAGCC[**T**/C]CTCTGACCCC |
| 9 | Ots28_11072994 | intergenic | GGGAGACTTAAAACAACCTCAAAA | ACCTGCAACCTTCTATTCAACAGT | CCATATGTCG[**C**/T]TTGT |
| 10 | Ots28_11073102 | intergenic | GGTGAGCCATTCATAACAATCTT | TGTTATCCTGGATCATTCAAGAGA | ACATTACTTT[**T**/A]CAAAAATATT |
| 11 | Ots28_11073668 | intergenic | CCTAAGAGGAGACGAGCATTACAG | GGTAAATCAACATATGACCACTCG | TACAGTTTCC[**T**/A]GTCTGA |
| 12 | Ots28_11075348 | intergenic | CATTTCAAAATTAGGAGGTTAGGG | AGATGAGAGCTGTGGCCTGT | GTGTGAAAGG[**G**/A]GAGAAGGGCT |
| 13 | Ots28_11075712 | intergenic | GCTTAAACAGCTGCTATTAGGACA | TAAGGATTTGTTGCCAGCTCTAAT | GAAAACTCTG[**C**/T]CCTG |
| 14 | Ots28_11077016 | intergenic | AAAATATGTGCAACATCCAATGTC | ACACAAGCTGGCTGAAGCTAAT | GTCAAACCAA[**C**/T]TTTGCCAAGG |
| 15 | Ots28_11077172 | intergenic | GTTTTGCCAGAGAGAATGTACAAA | TAGTGGTTAGAGCATTGGACTAGC | ACACACACAA[**G**/A]AGACACCCAC |
| 16 | Ots28_11077576 | intergenic | TGTGCGGAATTACTGATAATTGAC | GCTCTGCATTTTACAACACTGCT | GAAGGCC[**A**/G]AATAAAATTG |
| 17 | Ots28_11078636 | intergenic | CATTTGTCACGATTGTTACATTTTT | TAAACAACTAATTGACCGACGTTG | GCGATTAACC[**A**/G]ACATTTAATA |
| 18 | Ots28_11095755 | intergenic | CCAATGGTGATTTTAGAACCATTAC | AAAACAGAGTATGGATCAACAGCA | AG[A/**T**]GTTGAATGGC |
| 19 | Ots28_11143508 | intergenic | ACCTTTTAGCCAGTGACAACATTT | ATGCAAGAAACTCTCGACGATAG | TTCAC[G/**A**]TACGGCCCAT |
| 20 | Ots28_11147576 | intergenic | TTATATGAAGAGTGCCTTGGTCCT | CTGACAATTAATCTTGATGGTTGC | CCTTT[C/**A**]TTTTTGAAAA |
| 21 | Ots28_11160599 | rock1 | GTGCATATTTTACGTGGTTGAAGT | ATTCCATTTCACCCATATGAATTT | CTCTCTGCTT[G/**T**]CGTT |
| 22 | Ots28_11164637 | rock1 | TGATTTGACTTTTTGTGGTGTTTT | GTTCCAATCTGTTTTTGCTCTCTT | CTGGCGGGGT[C/**A**]TGGG |
| 23 | Ots28_11186543 | rock1 | GGCTTGCCTTTAGATAGAATCTTG | AAATCTCACAAGTCCAAAAACAAA | AAAGCTGATT[A/**T**]AAAA |
| 24 | Ots28_11201129 | rock1 | TGCGAGATTTATCTACTTGTCCAG | GGTAGTTTTGTACGCAATTGCTAA | ACTGAAGGAA[T/**G**]TTAAC |
| 25 | Ots28_11202190 | rock1 | GCTAAATGTAAATCGAGTGGCTGT | TACATGGGTCCTCTCAGTGTTCTA | CAAAAGTCTG[T/**C**]ATTTTCAAAA |
| 26 | Ots28_11202295 | rock1 | CTCAGTGTTCTAGAATGCAGGAAA | GTAGTCTAAGGCACTGCATCGC | CTCCCGGGTG[G/**A**]CGCA |
| 27 | Ots28_11202400 | rock1 | CCCTCCAAAAAGAAAACATTTGAT | AAATTGGCTAATCAAACACTGGTT | GACACACTCA[C/**T**]GA |
| 28 | Ots28_11202863 | rock1 | GAGGATGGATGAGACTTTTCAGAT | GCTCTTTACCGGGTTTATATGAAG | ATAAAAAATT[C/**A**]TGCGTGAATG |
| 29 | Ots28_11205423 | rock1 | TTAAATCACCCAGAGCTTGTTAGA | ACCTGACCTAGATAACAACCACAA | CCTGCACAC[A/**G**]TGTCAAACCG |
| 30 | Ots28_11205993 | rock1 | GCTGCTATTTCCGACCTTACAATA | ATCAAGACAAAACACTCACCAGAA | G[C/T]TATTAAAAGG |
| 31 | Ots28_11206740 | rock1 | ACTTTGAGGACTTACTCCTGTCCT | CTGGAGAAAGACAAGATGATGATC | CCTTCCCTCC[T/**C**]AGGGCAACGT |
| 32 | Ots28_11207428 | rock1 | TATACCTTTGTAGCATCCCTCTCC | CATATAAAGTGGACAGCGTTTGAC | GTTGGGAGCG[T/**G**]CCCAAAATGG |
| 33 | Ots28_11210919 | rock1 | AGTGCTCCATGCTGGAGTTT | GATGAAGCAGAAGGAGAGGCT | GACCT[C/**T**]AAGCAGTCAG |

**Table S2.** List of outliers from OutFLANK and BayeScan results for three populations of Chinook Salmon.

|  | Lower Columbia | Interior ocean-type | Interior stream-type |  |
| --- | --- | --- | --- | --- |
| BayeScan | Ots28_11207428 | None | None |  |
|  | Ots28_11210919 |  |  |  |
| OutFLANK | Ots_105105-613 | Ots_118205-61 | Ots_108820-336 |  |
|  | Ots_EndoRB1-486 | Ots28_11207428 | Ots_127760-569 |  |
|  | Ots_u07-53.133 | Ots28_11210919 | Ots28_11210919 |  |
|  | Ots_crRAD11620-55 |  |  |  |
|  | Ots_crRAD9615-69 |  |  |  |
|  | Ots28_11207428 |  |  |  |
|  | Ots28_11210919 |  |  |  |
| *Combined Total Removed* |  |  | N=10 |  |

**Table S3.** List of 185 final neutral SNPs used in analyses. The genome position of each SNP is listed based on genome assembly in NCBI (accession GCA_002831465.1; Narum et al. 2018). NA= unmapped SNPs.

| SNP | Chromosome | Genomic Coordinate | Forward Primer | Reverse Primer | Probe |
| --- | --- | --- | --- | --- | --- |
| Ots_myoD-364 | Ots01 | 7299784 | GTGTGTGTGTGTGTGTGTCATCGT | TTTACACATATACAAAAATGGTCCTCTATTGTCAT | ATCTTTTGTT[A,C]TTTCCTTG |
| Ots_redd1-187 | Ots01 | 9939318 | TTCTGGGTTGCCATACTCTTTCAAT | AGTTGAGACCTTCAGTTCTTAGGGTAT | CTGACAGC[T,C]GTTTTG |
| Ots_129458-451 | Ots01 | 11080640 | TGGGACCCACATAAAGCAACTG | GACATAAGACCCATTTAGCCCCTTTT | CATCTGGCA[A,G]TGCCTT |
| Ots_crRAD75581-70 | Ots01 | 27546510 | ACACATGGCTCGTCTGCA | GGAGCTCAGGGTGCAGGA | GAACTT[A,G]AAACACT |
| Ots_Prl2 | Ots01 | 40924770 | CCTGGTCTGTTTGTGATCAAGATG | GGTTAACTCAAATAGAACATACTCTGACACA | TGTATTGTTC[A,G]TTTAATG |
| Ots_107806-821 | Ots01 | 51993828 | TGCAGTGCTGAATTAGAGATTAATTTTTGTG | CTCCCTTGCTTTTGGTCATTGG | CAAAGAAAATC[A,T]AAATTT |
| Ots_crRAD69327-53 | Ots02 | 4708299 | GCCATTTGACCAACGGAGC | ACTCATACAGTATTTCCGCCTGT | ATAGGA[G,T]AATTGGA |
| Ots_TCTA-58 | Ots02 | 8993026 | ACCAGTACCTAAACGTTAGAAAGCAA | CGTTAGTTAGCTATGTCTGAAAGGCA | TGCCATGAA[G,A]TGCTAG |
| Ots_crRAD78968-46 | Ots02 | 23276932 | CCTGCTCTGTGTCTGGGC | GTGAAGACGACCCCGGTG | AGCAAT[C,T][C,A]CACAGC |
| Ots_nelfd-163 | Ots02 | 31069128 | CTCACTGCAAATCCAACTTCATCAT | CCACTACATCCTCATCCAAGGTT | CCACCAG[T,C]GTCATT |
| Ots_crRAD57376-68 | Ots02 | 54571149 | TGCAGGCATCATGCTTAATAACT | ACGTGACACAGGTCTGGG | ATAAAG[T,C]GTGTTAT |
| Ots_Est1363 | Ots03 | 8292351 | GGTGATTTTGCCACAGAGTAGAGAT | AGTGTTAAATGTAACTTGCATATACAGGCAAT | CATCCTGTC[T,A]TGTCTG |
| Ots_cgo24-22 | Ots03 | 12326537 | AGGTCCTCTGTCGCACCTA | GGAGGCGAGGTCTGGTG | CCAGATGA[A,G]CAACTTCAC |
| Ots_94857-232R | Ots03 | 27503532 | GGCACTCTCCCTGGCTAGA | CCCCATCACTTCTCTGGCTTTAAAT | CAGGATAATAAC[A,G]AACAAG |
| Ots_118938-325 | Ots03 | 43610834 | ATTTTCAAACAGGCATTTATCATTGGTGAA | GGTCTGTCCCTCATTCTTTGCA | AGAGATGCAAA[G,A]TGGAGTT |
| Ots_107285-93 | Ots04 | 5852987 | GCCCTTGTGACAATGCACTGTTATA | AACATACACCAATACTTAGGTCTAGACAGT | AAGTAACGTATCA[A,T]ATGGC |
| Ots_103122-180 | Ots04 | 16781457 | CAAACGCGCACTCACACACA | TCACAATGGTACGATTTTACGACTCAA | CATCAACAC[A,G]ATCTGC |
| Ots_crRAD24807-74 | Ots04 | 36172590 | TGCAGGAGAGCAGGGTAGA | CGTGCCTAACATCATGTGCA | ATGATA[A,T]T |
| Ots_u07-07.161 | Ots04 | 37799853 | GTCAACAAATGCAGGTAACATAAATGGT | GATGCAAACACCTGTGAAATTGTGA | TCAGTGACATAA[G,A]TTGTCCA |
| Ots_96500-180 | Ots04 | 45636897 | CAGGTCTGGTCTACATCGAACAC | GATCATGTCAGATAGGATGCTGAAAGT | AAAACAAAT[C,A]ATTTTTCG |
| Ots_HSP90B-100 | Ots05 | 6866692 | CACCTTAGTTCCACGCAACATG | CTGCGTGTATTGTAGTGGTGACA | TCTATGGTGT[G,A]ATTCATT |
| Ots_111681-657 | Ots05 | 8539947 | CTGAGCTTTTTCAACTTACTTGTTGGA | GGCGCAGCAGCAACTG | CGCAAAC[C,A]CCGAACC |
| Ots_crRAD66330-60 | Ots05 | 14208918 | ACTCTCCCAGAAGGATTCAGAGA | TCCCAAAGCATCCTGCCA | AGAGAG[G,T]GGTCAAA |
| Ots_U5121-34 | Ots05 | 20893356 | CCAGAGGTTAGATGGCCCTTT | CTGAGCCAGAACCACAAATTGAATT | AGGGTCTC[A,G]TGCTCCCT |
| Ots_HMGB1-73 | Ots05 | 27531907 | TGCTTCAGTGAAAATAAGCGTGAGA | GTCGAGCGGTATGAATACTTTCTGA | ACTGTATATGTTA[C,A]GTTTTC |
| Ots_u211-85 | Ots05 | 34253781 | TGGTGAGAGCAGCTTTAAATGTCTT | ACCCATTCTTCTGTCTGGTTTAAGC | CCCAAAGTC[G,A]AGTGTG |
| Ots_CirpA | Ots05 | 47621841 | GCTGTGATTGTGCTCTAAAGACATG | CTCCCACTTAGCATTCCTACCTT | CAGTT[C,T]TGTAATGCATT |
| Ots_u4-92 | Ots05 | 49324631 | ATCCAAGGAGCCCCATTAAAGATTT | CGTACCAGAGTTGTAGAAGCATCT | CTGTGTTGAATTTAAC[A,G]TAAT |
| Ots_127236-62 | Ots05 | 52450716 | TGGAGAACTTGCACTGAATGTGAAA | GCTGTTGGACCTTGACTTTAACAAATT | CTCTTATCTG[A,T]GTTCTGC |
| Ots_unk9480-51 | Ots05 | 58885154 | CAAATCAGAACAAAACCTCCCACAA | GGAAGTCTGTCTGAATGGTTGTCTT | TCCCA[C,G]AAACCC |
| Ots_SClkF2R2-135 | Ots05 | 60109411 | CCAAATACAGACCAGCTACTTGTGT | CTTCAAGTCCCTGAATAATGGTACGT | ATTCAAAGTC[A,T]AATTTT |
| Ots_109525-816 | Ots05 | 70296658 | GCCAGATAGTAGCGTACATCATGAG | CTCCCCATGTCCCTGAGTCT | ATGAGGC[G,A]TTCGGC |
| Ots_crRAD13725-51 | Ots06 | 10662607 | TGCAGGAGGAGGAAGGCA | AGAGCTGCCAGGTGGAGT | GAGGCC[C,A]CAGATTC |
| Ots_U2362-330 | Ots06 | 14699971 | AATGGGTAACAAAGAAATAGCTAGCTACTT | GACAGACCACAGTGAAGGTGAAA | CTGGGAAGA[T,C]TGTTTG |
| Ots_ZR-575 | Ots06 | 27576884 | GCCTACCAGAAAGTACCAATTGTGA | ACTTTTCACTGTCCTATTACAATTAGTATTTGTGATAT | CCGACA[C,T]AATTTTGT |
| Ots_94903-99R | Ots06 | 35539172 | CCGTCTGAGTAGGAGGATCAATACA | TTTGGATCCAGCTCTCCGTATAGA | CAAACCAG[C,A]AAACAT |
| Ots_FGF6B_1 | Ots06 | 37250687 | GAGACAAAGGTTTGCAGGTTCATG | GGGAGCCATGCACTAATATATTGGA | CTGTTATCAG[A,C]CCCAAAT |
| Ots_crRAD73823-60 | Ots06 | 53325628 | GCAGGAAGCAAAGTTCGGTG | AGCAACTCATCGCGTGGT | GCACGA[T,A]GCAGAAC |
| Ots_115987-325 | Ots06 | 66193572 | GGAGGTGTAGTGAAATGGGAAGAT | GCATTCAGTGAACCAGTAGTGCTAT | ATGCATAAAAGGT[A,C]ATTGTG |
| Ots_105385-421 | Ots06 | 73012849 | GACTGTCTTGGAACCGTTGCTA | TCCCGGAACACACCAATGTC | CTCCTGGG[T,C]ATATCG |
| Ots_mapKpr-151 | Ots07 | 5460143 | TGTTGTCTCGGACTGCATGAC | GAAGGCACAGAGATGAAGGACAT | CGTATGTGCA[A,T]TGCATG |
| Ots_mapK-3'-309 | Ots07 | 5480042 | GGCCACTGTCATAGAATTAGGCATT | CGTGACCCTTGTAACTGAAAAGC | ATGCTATTAAATGA[A,C]TATTC |
| Ots_ppie-245 | Ots08 | 23260130 | TGTTTTTGGTCATGTATTTTCTCTGCTATTTTT | GGACTGGAGCTGCTGAACATA | ATGTCTGAAAT[G,T]AAAGCC |
| Ots_101704-143 | Ots08 | 51040180 | ACTTCTTGAGCCAATCGGATGATG | CCAGAGATAAACTAGTGGAGGAGATCA | CTTAGACGTC[A,C]GAGGTC |
| Ots_103041-52 | Ots08 | 55669454 | ACCACCCACCTCCTCAGA | AGACAGAGAAAGTCGGGACACT | CATCCTG[C,T]TGGACCC |
| Ots_P450-288 | Ots09 | 19138835 | ATGTCAATATATTTCACTATAATGATTGGAAGCCA | CACTGAACTCGAAGCTGTTAGGA | AAAGTTGG[A,G]CAGTTGG |
| Ots_110201-363 | Ots09 | 25990000 | GTTTGGCTATTGAAATTATACATTAAAACATGTAGCT | CCATGGCATCCTGTAAAGAACAACA | TTTT[A,T]AAACTGGCATCCA |
| Ots_100884-287 | Ots09 | 34802127 | CGGAAGACCAGATTCTCCAAGAGTA | CGACCAAGTAGCGGCACTT | AACTACAATTC[A,G]CATATAT |
| Ots_hsc71-3'-488 | Ots09 | 37358812 | TGCATCCATTCATACCTGACCAATT | TTTGGTTAGGCACACGATAATTTGC | TTTCCAATG[G,A]TATAGATATGA |
| Ots_hsp27b-150 | Ots09 | 52471033 | TAGGAGTTGGAAAGACTGCACA | CCCATTGGTTCTTTGGTGTT | CGAT[C,T]TGGACCAGGCT |
| Ots_u1008-108 | Ots09 | 62354402 | GGATGACTCCTACTAATAGACGGATGT | AGGACAGGAAAGAAGCAGCAAATA | TGGTAAACCTGTTT[A,T]TTGGTA |
| Ots_Thio | Ots10 | 5625322 | TTTTAAAAATGGAGATAAACTCCTGACCTGAA | AATACCAAACCATGCCACTAATACCT | CAGTGTATTAGTC[A,G]TTCTTA |
| Ots_112820-284 | Ots10 | 16907470 | CATAGATGTTTATATGAAAAACCTCCCACTGT | GCATCCAAAAAGACGTGTGTGTTT | ACTCACACTC[G,A]AGTGACT |
| Ots_102414-395 | Ots10 | 20097469 | GCCTACTGATAAATGTATGACAGTAATGGA | CAATAACAAACAAGCTAGGAACAAAAGTGT | CACATAGTGTAGCT[T,C]TACTAC |
| Ots_crRAD18937-60 | Ots10 | 51017984 | GGCACAGCGACAGGAGTT | TGAGCTGGTGCGTCTGAG | CTCCTCA[G,A]GTGGGC |
| Ots_u07-57.120 | Ots11 | 16346192 | GGTTTGAGCCAATCAGTTGTGTT | CGGTCTAATGTCCATTGCTCATGTT | GTGACA[A,T]GGTAGGGGTTG |
| Ots_107074-284 | Ots11 | 16750220 | CCCACTTCCAGAGCCTGAA | TTTTCCATGGCTGTGTGTACTGT | CGTAGC[T,A]GCACCTG |
| Ots_108735-302 | Ots11 | 21337974 | CCTTTTTCTTATTAGTTTTACTTCCCCAGAGA | CAATTCCATTCTTGATTCTGTTTAACGGT | AACAAACAAC[G,A]CCTCATG |
| Ots_128693-461 | Ots11 | 29099828 | TCAATGTTCATCAATGCACTTCCTGTA | GCCTGCAGGAGAAGGTAGAGTTA | CTG[G,A]TACCCA |
| Ots_crRAD76512-28 | Ots11 | 32328258 | GCAGGGACAGGGCCCT | TGGTGCTGGGTGCTGTAC | TAAAAA[A,T]ATATAAA |
| Ots_crRAD36072-29 | Ots11 | 36019633 | TGCAGGACCAACTTTCTCAT | GGCTGACTGGTGAAGGGG | AACCTG[T,C]GTGATTT |
| Ots_crRAD61523-71 | Ots11 | 41149096 | GCCAAGTGATCAAGTGCTTGT | CCAGCAGTTCAGTTGCGG | CAGAGC[A,G]TGTGCTG |
| Ots_crRAD20376-66 | Ots11 | 46457753 | GGGAGGCAGGCAAAAGGT | GGTTCACCACCAGCCTTCT | GGGAAG[G,A]AGTATTT |
| Ots_crRAD47297-55 | Ots12 | 4878508 | CTCCCTGTTCGCTAGCCG | GGACGACCAAAGGTAGAACCC | TAGCCG[T,C]CACCGAT |
| Ots_NOD1 | Ots12 | 23568768 | GTGCTGCAGGAACCATGTG | CTGTGTGGACTGCTGTCTAAGG | CCAACG[G,C]CGACTTG |
| Ots_pop5-96 | Ots12 | 27353346 | CTCTTGCTACTTGCAGTGTATCTCA | AGTTTGAGGGCTCTATTCTGTCATG | CTGTTACTGG[A,G]C |
| Ots_u07-49.290 | Ots12 | 30314171 | GCTGAGGAAGGATTCTGTATTTGCT | TCGGACAGAGCGCATCC | CTTTCCC[C,T]GTGTTGGT |
| Ots_cox1-241 | Ots12 | 30421258 | CACTGAACTGTAAGCCATTGTGATT | GTAAATGTAGTATACAGTATAGGCATCGTAGGT | CACTAC[G,A]GTAAGACCAT |
| Ots_ETIF1A | Ots12 | 34678104 | TCTGAACTCACCAAAGGAACACTTG | GAGAGAAAAGGAGAAATGATTGCCATT | CTGAAGAAAA[T,G]AATATG |
| Ots_GCSH | Ots12 | 58052588 | GTTCTTTTTAATGATGACTACAGGTCTTTCAC | GCTACTTTACATAATACCATTTGAGCTGAGA | TATCTGG[G,A]CGGGCTG |
| Ots_brp16-64 | Ots13 | 4383923 | ACTCTGGGTCCAGGAGGTTTT | CTGACGAGACCATGCACCAA | AGTCAGC[A,G]TCTTTCA |
| Ots_crRAD27164-55 | Ots13 | 25285990 | GGAGGCTCTACGTAGGCCT | ACAATATCTGACACTGACTTGGTCA | AATTTG[A,T]ATGACCA |
| Ots_mybp-85 | Ots13 | 29231079 | CAAGGGATGTGACAAATTAATCAAACACATAA | AAGAGGTCTAATAAATCTCCAATGTAAAAACGT | AGCATGTA[G,A]TTTTG |
| Ots_hnRNPL-533 | Ots13 | 46705374 | TCTTTGATATTGAGCTCATAAAAGCAAGGT | TCCTTGTTCATCCATCAGGCATAAAA | TTTACCAGTTC[T,A]CACACAC |
| Ots_crRAD35313-66 | Ots13 | 65139272 | TGCAGGAAGAGTTCAGAGAAATCT | GCTCGTTGCAGGTAGAAATGT | TTTAAG[A,G]TGTAGTT |
| Ots_U2567-104 | Ots13 | 68622537 | CATAGTATAGTGATTCGAGTCTGGAGTCT | CGGGCTTTCTTAGGATATTTTCCTGA | GAGACT[G,A]TTGAGAC |
| Ots_crRAD57687-34 | Ots14 | 31889864 | TGCAGGGACGGGGCT | TGCTGTTGTCTTGGGTCTCTC | ACAAAT[T,G]AATTAAA |
| Ots_124774-477 | Ots15 | 15167502 | AGTTGTTCTTTTTATATTGTGTTTTTATTCCATTCCA | GCCAAATAAAAACAAAGCATGAACACA | CACCGCC[A,G]TCTGATA |
| Ots_112208-722 | Ots15 | 17637330 | CTGCATGAACGTTAACTCAAATAAAAGGT | AATGAGTTCTACTGACATTGTATACTAGAATAAGTATCA | TGTGAGG[G,T]CGGTCTT |
| Ots_102801-308 | Ots15 | 19971476 | TGGGACAGAGGTGGGAATTGA | CCCAAAGATGCTTAACTGAAGATGTG | AGGGACAGTTTC[G,T]CAGACG |
| Ots_117242-136 | Ots16 | 29333295 | GTGACAGGAGACAGAAAGAGACATT | TGGTCCTCCCTGTCTCTATCTACTA | AGCACATAAC[T,C]TGACCTC |
| Ots_unk526 | Ots16 | 29699840 | TCAAGACTGTGCTGTAGTTGTCTAC | CCTCCCCCTTTTCCACATCAG | CATTCCAG[T,C]CTGAAAC |
| Ots_SWS1op-182 | Ots17 | 7365688 | TCAAAGACATCGAACACAAGAACGA | GCAGGTAAATTCAAACGTCATCATAAGAA | ATGTACTTTAACG[A,T]TTCATTT |
| Ots_vatf-251 | Ots17 | 7664045 | CTTTTCGGGTTATTCATGCTGTTGT | GCAAGCATTTGAAAAACAGACTGGAT | AGACCACAAGATA[CA,-]GTACC |
| Ots_crRAD16540-50 | Ots17 | 9728418 | TGTGTATTCGTCGACCGGA | TCACCTGACCAAAGCACTGG | ATTAAA[C,T]GTCTGGA |
| Ots_crRAD2806-42 | Ots17 | 14222969 | GCAGGGGCAGACTGAAGG | ACTTCATGCCAATCTCACTAAACA | GTTTGG[C,A]ATAAAGT |
| Ots_crRAD22960-32 | Ots17 | 14223038 | ATCAGGTCTGGGGCGACA | TTCACCTCTGCCATCGCC | CGACAC[C,T]ACTTACA |
| Ots_crRAD33491-71 | Ots18 | 2699387 | CAGTTCGCTTCTCCAGGGA | TGTGGGTAGCAGACTGACG | GAGAGC[C,T]GAGCTTT |
| Ots_crRAD12037-39 | Ots18 | 10327070 | TGCAGGAACTTGCTATGCT | TGTGGAAAAAGTCAAGGGGTCT | CATTCA[A,G]AAAGTAT |
| Ots_123921-111 | Ots18 | 25247852 | TCGCTAGGCAGAAATATAGGGTTCT | GAGCATGGCGCTTGCA | CTAAATGGCA[T,C]ATATTAT |
| Ots_S7-1 | Ots18 | 31651991 | TGCCATCATAAACAACCTAACAAGTAACT | CCTGGTTTAAAAACGGCCAACTG | CAGGAGATA[A,G]GGTCGCA |
| Ots_pigh-105 | Ots18 | 35641063 | GCATTACTAAAAACTGGTGTGTGGAA | GTTTGGAATGTTTCTCTGATTGTGTTAACAA | ACCTGAAAATATAT[A,T]TTTTT |
| Ots_CD59-2 | Ots19 | 9312796 | CATGTTACCCAGCTAAAAGTCTATAGCA | TGTTTATCTCTGAGTGAAAAAGGTGTGT | CTAAAATGTCAT[G,A]TAAATAT |
| Ots_RAG3 | Ots19 | 34427967 | CATTTCCACGAAAAGCCAGATGAC | ACAGAATAAAGTATCTTCCTCTTACATCACTACTAAT | CTCTACA[G,A]TATG |
| Ots_110381-164 | Ots19 | 35228582 | CTCTTGTTTGCTATGGGAGATGTAGT | CCGTATCCTAAACCCTTCACTGTT | TTGCGTC[T,C]TCTCCC |
| Ots_95442b-204 | Ots19 | 39212513 | GTCTCTCTCTCTTTGCATCATTACACT | GGACTCTTGAGCTGTCTGGCTATAT | TGGTTCCCC[A,T]AATTT |
| Ots_crRAD20262-46 | Ots19 | 46397112 | CCTCTGCTGAGTTTGAGGGG | TGAGCAGAGCCTATGAGGACT | GGTTAC[A,G]CCCCAAA |
| Ots_108007-208 | Ots20 | 4971142 | CAGGCTTGTGTTAAGTAGGGAGAAA | CATTGGACAAGACCGGGTAGTC | TTTCACTTAATTT[T,A]AAAATG |
| Ots_crRAD20887-70 | Ots20 | 7378067 | CTGCTTGTAGCCGTTCAGC | AGAACACATCTGGCCAGGT | GAACTC[G,A]TCGTTGG |
| Ots_IL11 | Ots20 | 20625334 | CCTCCAGATGAGACCCACTCT | CAAAATGGTGCTCAAACGACTTCA | AGCTCCA[T,C]GCGGACT |
| Ots_crRAD44588-67 | Ots20 | 25774788 | CGCAAGTCAGCAGGGTGA | TGGGGTTTTAGGCTGGGT | GTGAAC[C,T]AATCAAT |
| Ots_Est740 | Ots20 | 39254576 | GGACTCGTGCTTGAGGAAGATG | TGCATGGCTCCAACTCCTT | CTGGATGGA[A,G]CCGTTAG |
| Ots_AsnRS-60 | Ots20 | 41784960 | CCGACGCCTCACTGAGT | TGGTTTTTCAGGTCATGGTTTCCA | AGTCCC[T,C]GACCAGC |
| Ots_AldB1-122 | Ots21 | 5255056 | GCCATGGAGGACTGGATGA | GCCACCACTACTTGCTGAGAAAATA | TGTTGG[C,T]GAAGTGGGT |
| Ots_105132-200 | Ots21 | 9585379 | CGATGTACTGAGGGCAGTGT | GAGTGGAGTTCCTTAATAATCATTGACCTT | CAAGAGTGG[C,A]ATAAAA |
| Ots_101554-407 | Ots21 | 9784111 | TGAAAGATATCAATTGTAGTAGTGGTGGTG | ACACGCCAGTCCACAAGT | ATGGAGGATT[G,C]TGGTTGT |
| Ots_crRAD60620-51 | Ots21 | 24004835 | CAGGCAGTCACTGAGTCCG | TTTGAGCACCGTTTCCGA | GTACGG[A,G]AAAAACA |
| Ots_E2-275 | Ots22 | 5457012 | GGTGCCACTTTAGTATAGCTGCTTA | CCCTACCCCCTGTGTTCCA | CCCCA[T,C]ATTGCTG |
| Ots_parp3-286 | Ots22 | 9505120 | AGTCAGTGTTGGTGTAGTGAAGAGA | CATTTGTGGAGTGTTTATTGAACAGTAACA | ACAAGTGG[T,C]GTTTCA |
| Ots_CD63 | Ots22 | 13378891 | TGCATGTTTTCTAACTGTGTTTTTGTGT | TGAATGCCCCCCATCAACA | ATCATGGG[A,C]ATCATAT |
| Ots_110551-64 | Ots22 | 14420914 | GAGTGGTCAAGGTTTCAGTTTCTG | GAAATGGACAGACACAAGGTCAAAC | ACGCTC[G,T]GAACATT |
| Ots_u1002-75 | Ots22 | 15468014 | CCGCCTTTCCCACCTTCTC | TCAAACGAGAACACACTAAGGTTGT | TGGCCCTTAC[A,G]CTATC |
| Ots_113457-40R | Ots23 | 9447490 | CCCAAGTGGTGAGTGTCAGT | ACTACAACAGGTGTTGATAATAGAATCATTCTC | CCCTATTCTC[C,T]AATCCATAT |
| Ots_PEMT | Ots24 | 15921271 | AGAGCATTCAATTTAAAAGCTGAAAACGA | CTTTGATCCCTGCTTGCAGTATTTT | TGCATTG[C,T]TAAGACTTG |
| Ots_106747-239 | Ots24 | 17321046 | ATCGAGGATGCCTCAAAGACATC | GTTAGACCCACCACCAGTCATC | CCCGC[G,T]GTGAGTAT |
| Ots_GTH2B-550 | Ots25 | 27327552 | CACAGGAAGGACGTGTTTTGATG | TGACTACCCGTTGTACCAATGAAC | ATAACAT[C,G]TGCAGCATTAA |
| Ots_112876-371 | Ots26 | 29108047 | GCCTACAGCAAATTCAGCTACACAT | TGGACCTTCAATCATCACAGCTT | CATCACAAC[G,T]ATGTGTG |
| Ots_110064-383 | Ots27 | 8141061 | AACAAAGAATGTTAAACACCAAACAGGAA | GTGCAAGGGACCTAGCTAATCC | ACGTAATGAAC[G,A]TTAGCT |
| Ots_RAD4543-52 | Ots27 | 12866217 | TCTTTGGACTGTGTATACCAGGTGTA | GCCAGATGCTGTGTGTGTTT | TACATA[T,C]GACTAATGAAA |
| Ots_U212-158 | Ots27 | 14522192 | CCCCATATGAGACGCTACAGTAATG | CAAATGCCCTCTAAGCAGACCTT | CTGGAA[G,A]AAGGCCTC |
| Ots_trnau1ap-86 | Ots28 | 17646942 | GGACAAGTTGAAACAGATCAGGAAGT | GCCACTGGATACCATCACTTCAAA | TCCCTC[C,A]TTTTTCC |
| Ots_OTSTF1-SNP1 | Ots28 | 25845386 | CGGACAAAGAGCTACAGAAATGC | CGTCCCTCTTCACGCATGA | CGCCAC[C,A]TTGGCT |
| Ots_97660-56 | Ots28 | 34830271 | TTCCCTAATCTGACGTACTACCAACT | CGCCACTGACGTTCATTCCA | ACGAGAC[A,T]GATATTC |
| Ots_117432-409 | Ots29 | 10328032 | TCATCAAAACATGCCTCTTCTGTGT | TGTTGAACCTGTCACTCTGTCTTC | ACTTTGCTC[T,C]ATAACAG |
| Ots_U2446-123 | Ots29 | 11728129 | CTGGTCTGTGACGTCAAAATGATG | AGCTAGACCAGGCCATTTGAG | CTGCAACTC[G,T]ACGCAAG |
| Ots_96222-525 | Ots29 | 12489280 | GCTCTTGCCCATCTGTAGGAT | GGCGCAACATATGTATTAAGCAACT | AGCTAATTTTAA[G,A]TTCTC |
| Ots_111084b-619 | Ots29 | 19749885 | TTGTGGAATTACACCTTCAGAGTTCAAT | GCCTGTTTGGCTTTCTTAAACTGAT | TCCATGGAAAC[G,T]GACAAT |
| Ots_crRAD57520-66 | Ots30 | 1332375 | ACAGAGCTGTGTCTACCAGA | ACCCTCTCTTGGCCTTGC | TTTTTG[T,G]TCAAAAG |
| Ots_P53 | Ots30 | 2417591 | GGAACTTCCTCTCCCGTTCTG | GCACACACACGCACCTCAA | TGGGTCG[G,A]CGCTC |
| Ots_u07-18.378 | Ots30 | 6130660 | GGAAACCAGCTAGGATTCAGGAA | CGTTATATGGTTTGCTTGTTTGCGATA | TATGTAGAGGC[T,A]AGTTA |
| Ots_crRAD25367-50 | Ots30 | 8042526 | ACTGCAGGCGTCATGCTT | TGGACAAAAGACCACAGGCT | GTATAT[T,G]TAGAATG |
| Ots_130720-99 | Ots30 | 17928099 | CGGTCATTGTAAATGTCAACGGTTT | TGCTTGCATGTTCTTGGTGTAGTAA | CTGTC[T,C]CATTCCC |
| Ots_TAPBP | Ots30 | 34770823 | TTTCTCATCCTTCTCTCTTCCAGTCT | GGACAAACCAGCACTCCAGAA | CAG[C,T]TGTCCAGTTCTG |
| Ots_crRAD255-59 | Ots30 | 40093591 | TGCAGGAGCTGTGATGGG | GTACGGAGCGTCACTGCT | AACTGT[T,C]CAAACCC |
| Ots_ntl-255 | Ots31 | 15370000 | TGCAGTTACAAGCCTAAGACAATCT | CAACTAAAGTAACACACCAGCAACTG | AT[T,A]CTTCCTCCACAATTG |
| Ots_unk1104-38 | Ots31 | 21291286 | TAACCATGACTTCTATCAATCACCCC | CCTCCATACATCGTCAAAGCTGTA | CACTAAGGATTAC[G,A]TTACG |
| Ots_DDX5-171 | Ots32 | 5948580 | ATGACCAATTGAAGAGTTCTTCCGT | CAAAGCCAAACGTCACATTTACACT | CATAATTGAAC[G,A]ATTTCA |
| Ots_112419-131 | Ots32 | 7853251 | GTGGGTAATCGATGCCAAAGAGAT | TGGCAGTGTTTTCAACTAGCTTTG | AGCGAC[T,A]TGATTATC |
| Ots_sept9-78 | Ots32 | 9390357 | GTCGATTACCGTTAGCTTCATCCT | ATTTCTCCTGTGTCTCTCTCTGTCT | CTCTTC[G,A]ATGTCTAGACA |
| Ots_afmid-196 | Ots32 | 17096334 | CGTGGAGTAGGTGGTTACAGTTTAT | CTCGTAACAAGCTACTGTAGTGTACT | AAGTCAAAGATC[C,G]TATTAAA |
| Ots_Ostm1 | Ots33 | 4569955 | CCAGCCCCGTAACACACAT | GAGAGGAAGCAGAAAGGTCGTTTAA | CCGTGGTATT[G,C]TTTCAA |
| Ots_NAML12-SNP1 | Ots33 | 9777802 | TGCCACCTCAGTTTTAGTGTTATATCC | AGCGCCAACCTGTCACT | CCATTTTCA[T,C]TCTTTTG |
| Ots_TGFB | Ots33 | 29760263 | GCCTCACATTTTACTGATGTCACTTC | GAGCAGATCTCTTCAGTAGTGGTTT | AGCCTAG[C,T]TCTCGGAAG |
| Ots_TLR3 | Ots34 | 7365214 | TGCACCTGCGAGAGCAT | CTGGCGTTTGTTCCGTTCAG | CTGTGGTTTGT[G,A]GCGTG |
| Ots_113242-216 | NA | NA | GAGGCCTAATGTCTCTTGTGACT | GACATCTTCAACAAGTGTTCATTCACC | TTACCAAC[G,A]GAGAACC |
| Ots_128757-61R | NA | NA | CGTGTCCGGCTTCTTTTATTTCATT | GATGGGTATGTTAATCATATTACCAGCGTAA | TGTGCATTT[T,C]CCCC |
| Ots_Cath_D141 | NA | NA | CACTTGTTCTGCACACTACTTGTC | CACACATGGATTTTGCCTGTCTAAA | TGGGAAGCA[A,G]TCAA |
| Ots_EP-529 | NA | NA | GCCCTGCCTGCAACTTC | GAAACCAACGTCTTGATGTAGACCTA | CAGTGTCAT[T,C]TTCGGC |
| Ots_MHC2 | NA | NA | GTCCTCAGCTGGGTCAAGAG | GTAGTGGAGAGCAGCGTTAGG | CTGGAGCGT[T,G]TCTGTA |
| Ots_u6-75 | NA | NA | GAAAAAGTAAAGTAAAAGTAAAGTATTATACCACTAAAGACAAT | GATCCACACTGTTGGTCTACTACAA | TTAGTCAACTGTT[G,A]TTTTT |
| Ots_110689-218 | NA | NA | GTATAAACTAGAGTCCAGTGTTATGTTAATGTCTT | CATGGCAGACAACAGTAGAGAATATGA | ACCAATCAATT[A,C]ATTATT |
| Ots_106499-70 | NA | NA | ACTCTATCATCGGCAGGACCAT | ACCGTAAGTGTGGTTGTGTTCATTA | CATTTTTCAGAATT[G,C]TATTC |
| Ots_131906-141 | NA | NA | GGCTCGAACCACCCAGTTTA | TGCCCAACTGGTTTGCAATC | ACGGTTTACACTCC[T,A]ATTA |
| Ots_HFABP-34 | NA | NA | CAAGAACACCGAGATCTCCTTCA | TCGGCGGTGGTCTCG | TCGAACTCC[G,A]CTCCTAG |
| Ots_104063-132 | NA | NA | GCGTTACTGGTGTTATAAACGTTAGC | GTTTATTTAATTATGAAGGACGATGTTGAAGTCA | CTTTCGTCCTTA[G,A]CACATAG |
| Ots_crRAD74766-28 | NA | NA | GCTGACCACCGACCACAG | AGCTCTGCAGTAACAATGGGA | AGACTG[G,A]TAAAAGA |
| Ots_aldb-177M | NA | NA | GCGATCAGGTGACGCTAAAATGA | AGGAAGGTGATGCCTGAGAGA | CCAAATTGCTT[T,A]ACCC |
| Ots_FGF6A | NA | NA | CTTGTGCGCACCTTGCA | TCAAAAATGTCTATCCAACAAATACTCTGAAAAATATTG | CACGATTAGCAAT[G,T]AACAA |
| Ots_hsc71-5'-453 | NA | NA | GTACGAAGTTGCGCCTTGTC | TTGAGAACATGTGGTAATTAACTACAATGACTAA | GCCTGAGGTG[G,A]CAAA |
| Ots_MHC1 | NA | NA | GTCCACATTCTCCAGTACATGTATGG | CAAACCCCTCTGTCTGTTCAGT | TCATCATCCC[G,A]TGAGCAG |
| Ots_Tnsf | NA | NA | GCCAATACGGGTTCTGAACTGT | CGGAATAGTCATAGTAGGGCTCGTT | TGCTCCAG[A,G]TCTC |
| Ots_104048-194 | NA | NA | CAGCTGCTGCAGTCAATGAG | GCTCCTTACCAGTGTTGTCAGT | C[C,T]GCCACCGCCACCAC |
| Ots_111312-435 | NA | NA | ATGTGACCTCTAGGTATGAGT | ATGTGATCTCTAGGTATGAG | ATGTGA[T,C]CTCTAGGTATGAG |
| Ots_crRAD26541-47 | NA | NA | TGCAGGAGAAGCTGACTGAC | GCAGCTTTGGAGGGTCGT | CTGCTC[G,A]ACAGAGA |
| Ots_crRAD28677-65 | NA | NA | CTTGCGCGGCAGTTGAAC | TGTGTCCTCCGAAACATGACC | CTTC[C,T]GGGTTAAGAGGA |
| Ots_crRAD30341-48 | NA | NA | TGCAGGCTGACTTGGGTA | TAACCCGGAAGCCAGCTG | G[AG]CACA[A,T]TGGTGCA |
| Ots_crRAD33054-62 | NA | NA | CGACCATGGCCCCCAATTCAT | GTGAGGCTGACCGTGACC | CACGGG[A,T]ATGGACA |
| Ots_crRAD42058-48 | NA | NA | AAGCTATGCAGGCGACGG | GTGCACAATTGGCCCAGC | GAGCCA[A,T]GTAAAGC |
| Ots_crRAD5061-27 | NA | NA | AGATGCAGGAGGCTCTGGA | AGGACTGGAGTGTGATAGCAC | ATTAGG[T,C]AT[CT]TTAA |
| Ots_crRAD57537-24 | NA | NA | TGCAGGGGGACAAGAGAGA | CGCTCATAGCTTGTTCTCCA | GAGCCC+[A,C]TGAGAAA |
| Ots_u07-17.373 | NA | NA | TTGCCCATTCACCATCGGAAT | CACTGCACCAAATCACTCTTTCAAT | [A,-]CACATACACATTTGAATG |
| Ots_OTDESMIN19-SNP1 | NA | NA | GGTCTGTCTGTCTGTCTATCTGTCAATG | TGTGTGTCTTTGTTCATTCCTACCA | CCAGTCAT[G,T]GGTCATT |
| Ots_unk3513-49 | NA | NA | TTTGAGTGAGTCACTGCACCAA | CAGCTCCACAGTGTCACCAT | AGTGC[G,A]AAGAACC |
| Ots_105407-117 | NA | NA | TGTGTACATCCGCGTAAATATTGAAGATAA | CTGTGAGCTGCTGCAAACC | CAGGTTAGGA[A,T]TGGTTG |
| Ots_112301-43 | NA | NA | GCATGGCTGCCCTAGAACA | TCAGAACATTTCCTTCAGCTTCGT | CGTCGC[A,G]TTCAGC |
| Ots_GDH-81x | NA | NA | CTTTTCTGAATTAGTGCTGTGCTTGT | CCAACTTCTTCAACTCTGTCAGTGA | TGTTAC[G,-]GGACATACT |
| Ots_102213-210 | NA | NA | CATTCCATGACAATGATTGAAATCTAAAAACAC | GAGTATCTCAATTGCAACACTATGGTATGT | ACAGTAAGAG[T,C]ATTAAT |
| Ots_OTALDBINT1-SNP1 | NA | NA | CGCTGGGCATGGATGAGT | GGCCAACACTGCTACTTCCT | CTGTTGT[A,G]TTTTCTC |
| Ots_102457-132 | NA | NA | CCAGCAGAGACTGGGTTCAC | TTCCCTACCGGCGAAACC | TGGGGC[A,G]ACGCACAATTGGCT |
| Ots_unk7936-50 | NA | NA | ATGGGTTGGGATTATGGTTCATTGT | CAAAATGGTTACTTGCATAGTCTTTTGT | AGACATGTAGCTAT[G,C]TAGGTAA |
| Ots_u202-161 | NA | NA | CACTTTTGACTTTACATGGAACTTAACTCAT | GGGACTTCACTTTCTACAAACATGTCA | AGCTAGTGC[T,A]TAGCAGCTAA |
| Ots_U5049-250 | NA | NA | CAATGTCTAAAGTAATGGTGGTATTCTTGC | TCTTTGACACACCATCTGCCAATT | TGGAATG[G,T]GTAAGGTGTA |
| Ots_U2305-63 | NA | NA | TGTCATCTCTATTGCAATCTCAGTAGATTTCTAT | CCAGGTCGTCTTTATTGCAGATTATCA | AATGTCATA[T,-]AGAAATCTAC |
| Ots_unk1832-39 | NA | NA | GAAACGTCTATGCTGTCCCCTTTAA | CTGCAGTATTAGCTCTAGTTGAATCCA | CACCACTA[G,A]AACTCTC |
| Ots_126619-400 | NA | NA | GGATGGTTGTCATTTCTCTGCAAA | CCGGGATACAATAATAATATTTGGTTAAGAGTTTTTT | AAAGTTCTAG[A,G]AATAATT |

**Table S4.** Haplotype block combinations with frequencies for each of the three lineages of Chinook Salmon. Haplotype blocks follow designations in Haploview which included a single block of 32 SNPs for both the Lower Columbia and Interior ocean-type populations. The Interior stream-type population included two distinct blocks with 17 (block 1) and 16 SNPs (block 2).

|  | Block | Haplotype | Frequency |
| --- | --- | --- | --- |
| *Lower Columbia* | 1 | CGCAGGGCTAAATTAGGAGCGCATCGCGTCTC | 0.19 |
|  | 1 | TAAGACATCTTGCCGAATAATATGCATGTCGT | 0.14 |
|  | 1 | CGCAGGGCTAAATTAGAAGCGCATCGCGTCTC | 0.08 |
|  | 1 | TAAGACATCTTGCCGAATAATATGCATGCCTC | 0.07 |
|  | 1 | TAAGACATCTTGCCGAATAATATGCACGCCTT | 0.07 |
|  | 1 | TGCAGGGCTAAATTAGGAGCGCATCGCGTCTC | 0.06 |
|  | 1 | TGCAGGGCTAAATTAGAAGCGCATTGCACTTC | 0.06 |
|  | 1 | CGCAGGGCTAAATTAGGAGCGCATCGCGCCTC | 0.04 |
|  | 1 | CGCAGGGCTAAATTAGGAGCGCATTGCACTTC | 0.04 |
|  | 1 | TAAGACATCTTGCCGAATAATATGCATGTCTC | 0.03 |
|  | 1 | CGCAGGGCTAAATTAGAAGCGCAGCACGCCTC | 0.03 |
|  | 1 | CGCAGCATCTTGCCGAATAATATGCATGTCGT | 0.02 |
|  | 1 | TAAAGGGCTAAATTAGGAGCGCATCGCGTCTC | 0.02 |
|  | 1 | TAAGACATCTTGCCGAATAATATGCATGTCTT | 0.02 |
|  | 1 | TAAAGGATCTTGCCGAATAATATGCATGTCGT | 0.02 |
|  | 1 | TGCAGGGCTAAATTAGGAGCGCATTGCACTTC | 0.02 |
|  | 1 | TGCAGGGCTAAATTAGGAGCGCATCGCGCCTC | 0.01 |
| *Interior ocean-type* | 1 | CGCAGGGCTAAATTAGGAGCGCATTGCCACTT | 0.32 |
|  | 1 | TAAGACATCTTGCCGAATAATATGCATCGTCG | 0.27 |
|  | 1 | TAAGACATCTTGCCGAATAATATGCATCGTCT | 0.17 |
|  | 1 | TAAGACATCTTGCCGAATAATATGCATAGTCG | 0.05 |
|  | 1 | CGCAGCATCTTGCCGAATAATATGCATCGTCG | 0.02 |
|  | 1 | TAAGACATCTTGCCGAATAATCTGCATCGTCG | 0.01 |
|  | 1 | CGCAGCATCTTGCCGAATAATATGCATCGTCT | 0.01 |
|  | 1 | CGCAGGACTAAATTAGGAGCGCATTGCCACTT | 0.01 |
|  | 1 | CAAGAGGCTAAATTAGGAGCGCATTGCCACTT | 0.01 |
|  | 1 | CGCAGCATCTTGCCGAAAGCGCATTGCCACTT | 0.01 |
| *Interior stream-type* | 1 | TAAGACATCTTGCCGAA | 0.79 |
|  | 1 | CGCAGCATCTTGCCGAA | 0.02 |
|  | 1 | CGCAGGGCTAAATTAGG | 0.17 |
|  | 2 | TAATATGCATCGTCTT | 0.23 |
|  | 2 | AGCGCATTGCCACTTC | 0.54 |
|  | 2 | TAATATGCATCGTCTC | 0.13 |
|  | 2 | TAATATGCACCGCCTT | 0.02 |
|  | 2 | TAATATGCATCGTCGT | 0.02 |
|  | 2 | TAATAAGCGCCGCCTC | 0.01 |
|  | 2 | TAATATGCATAGTCGT | 0.01 |
|  | 2 | AAATAAGCGCCGCCTC | 0.02 |
|  |  |  |  |

**Table S5.** GWAS results for 33 SNPs predicting arrival timing in Chinook Salmon using MLM as implemented in GAPIT. P-values are represented as Benjamini Hochberg FDR-corrected p-values; *p<0.05. SNPs that were significant in all three populations are highlighted in bold.

| SNP | Lower Columbia | Interior ocean-type | Interior stream-type |
| --- | --- | --- | --- |
| **Ots28_11008798** | 1.66E-10* | 1.88E-22* | 4.47E-20* |
| **Ots28_11023212** | 2.25E-16* | 7.29E-22* | 9.97E-20* |
| **Ots28_11025336** | 2.25E-16* | 2.10E-21* | 9.97E-20* |
| **Ots28_11033282** | 1.54E-16* | 2.10E-21* | 7.24E-20* |
| **Ots28_11042701** | 1.54E-16* | 2.10E-21* | 1.86E-19* |
| **Ots28_11062192** | 3.74E-18* | 7.89E-24* | 1.71E-25* |
| **Ots28_11070757** | 2.48E-19* | 4.32E-23* | 3.21E-26* |
| **Ots28_11071377** | 2.03E-20* | 5.01E-24* | 3.21E-26* |
| **Ots28_11072994** | 2.03E-20* | 5.01E-24* | 3.21E-26* |
| **Ots28_11073102** | 2.03E-20* | 5.01E-24* | 2.65E-26* |
| **Ots28_11073668** | 2.03E-20* | 5.01E-24* | 3.21E-26* |
| **Ots28_11075348** | 2.03E-20* | 5.01E-24* | 3.21E-26* |
| **Ots28_11075712** | 2.03E-20* | 5.01E-24* | 3.21E-26* |
| **Ots28_11077016** | 2.03E-20* | 5.01E-24* | 3.21E-26* |
| **Ots28_11077172** | 2.03E-20* | 5.01E-24* | 3.21E-26* |
| **Ots28_11077576** | 2.03E-20* | 5.01E-24* | 3.21E-26* |
| **Ots28_11078636** | 6.99E-12* | 7.92E-23* | 2.90E-26* |
| **Ots28_11095755** | 1.34E-19* | 3.76E-25* | 1.12E-26* |
| **Ots28_11143508** | 7.04E-20* | 3.76E-25* | 4.12E-28* |
| **Ots28_11147576** | 2.03E-20* | 3.76E-25* | 4.12E-28* |
| **Ots28_11160599** | 2.03E-20* | 3.76E-25* | 4.12E-28* |
| **Ots28_11164637** | 2.03E-20* | 1.31E-23* | 4.12E-28* |
| **Ots28_11186543** | 2.03E-20* | 1.02E-24* | 4.12E-28* |
| **Ots28_11201129** | 4.04E-20* | 1.31E-24* | 4.12E-28* |
| **Ots28_11202190** | 0.0002247* | 7.07E-24* | 2.20E-27* |
| **Ots28_11202295** | 4.04E-20* | 1.31E-24* | 4.12E-28* |
| **Ots28_11202400** | 3.81E-15* | 1.72E-24* | 4.12E-28* |
| Ots28_11202863 | 0.00433619* | 5.61E-06* | 0.96041754 |
| **Ots28_11205423** | 0.00015259* | 5.01E-24* | 4.12E-28* |
| Ots28_11205993 | 0.82471815 | 5.01E-24* | 9.30E-28* |
| **Ots28_11206740** | 0.00015259* | 5.01E-24* | 4.12E-28* |
| Ots28_11207428 | 3.25E-09* | 4.32E-18* | 0.96041754 |
| **Ots28_11210919** | 2.07E-12* | 2.22E-18* | 1.89E-13* |

**Table S6.** GWAS results for 33 SNPs predicting fitness in the Interior stream-type population using MLM and BLINK as implemented in GAPIT for males, females, and sexes combined. P-values are represented as Benjamini Hochberg FDR-corrected p-values; *p<0.05.

|  |  | MLM FDR-Adjusted P-values |  |  | BLINK FDR-Adjusted P-values |  |
| --- | --- | --- | --- | --- | --- | --- |
| SNP | Males | Females | Combined | Males | Females | Combined |
| Ots28_11095755 | 0.012* | 0.128 | 0.009* | 0.24923357 | 0.18788315 | 0.99573803 |
| Ots28_11186543 | 0.012* | 0.128 | 0.009* | 0.24923357 | 0.22334792 | 0.99573803 |
| Ots28_11202400 | 0.012* | 0.168 | 0.009* | 0.24923357 | 0.35766668 | 0.99573803 |
| Ots28_11202295 | 0.012* | 0.128 | 0.009* | 0.24923357 | 0.22334792 | 0.99573803 |
| Ots28_11143508 | 0.012* | 0.128 | 0.009* | 0.24923357 | 0.18788315 | 0.99573803 |
| Ots28_11147576 | 0.012* | 0.128 | 0.009* | 0.24923357 | 0.18788315 | 0.99573803 |
| Ots28_11164637 | 0.012* | 0.128 | 0.009* | 0.24923357 | 0.18788315 | 0.99573803 |
| Ots28_11201129 | 0.012* | 0.128 | 0.009* | 0.24923357 | 0.18788315 | 0.99573803 |
| Ots28_11206740 | 0.012* | 0.128 | 0.009* | 0.24923357 | 0.18788315 | 0.99573803 |
| Ots28_11160599 | 0.012* | 0.128 | 0.009* | 0.24923357 | 0.18788315 | 0.99573803 |
| Ots28_11205423 | 0.012* | 0.128 | 0.009* | 0.24923357 | 0.18788315 | 0.99573803 |
| Ots28_11202190 | 0.013* | 0.128 | 0.009* | 0.254705 | 0.18788315 | 0.99573803 |
| Ots28_11205993 | 0.012* | 0.288 | 0.013* | 0.24923357 | 0.62358723 | 0.94305565 |
| Ots28_11202863 | 0.139 | 0.893 | 0.265 | 0.25504467 | 0.96331593 | 0.94305565 |
| Ots28_11210919 | 0.204 | 0.555 | 0.268 | 0.26897361 | 0.96331593 | 0.99573803 |
| Ots28_11042701 | 0.204 | 0.555 | 0.296 | 0.62975676 | 0.96331593 | 0.17054148 |
| Ots28_11062192 | 0.204 | 0.555 | 0.343 | 0.32967167 | 0.96331593 | 0.17054148 |
| Ots28_11033282 | 0.204 | 0.555 | 0.343 | 0.52245291 | 0.96331593 | 0.17054148 |
| Ots28_11008798 | 0.204 | 0.555 | 0.343 | 0.52369069 | 0.96331593 | 0.17054148 |
| Ots28_11023212 | 0.204 | 0.555 | 0.343 | 0.52145393 | 0.96331593 | 0.17054148 |
| Ots28_11025336 | 0.204 | 0.555 | 0.343 | 0.52145393 | 0.96331593 | 0.17054148 |
| Ots28_11070757 | 0.204 | 0.555 | 0.343 | 0.26897361 | 0.96331593 | 0.15538387 |
| Ots28_11072994 | 0.204 | 0.555 | 0.343 | 0.26897361 | 0.96331593 | 0.15538387 |
| Ots28_11075712 | 0.204 | 0.555 | 0.343 | 0.26897361 | 0.96331593 | 0.15538387 |
| Ots28_11077016 | 0.204 | 0.555 | 0.343 | 0.26897361 | 0.96331593 | 0.15538387 |
| Ots28_11077576 | 0.204 | 0.555 | 0.343 | 0.26897361 | 0.96331593 | 0.15538387 |
| Ots28_11071377 | 0.204 | 0.555 | 0.343 | 0.26897361 | 0.96331593 | 0.15538387 |
| Ots28_11073668 | 0.204 | 0.555 | 0.343 | 0.26897361 | 0.96331593 | 0.15538387 |
| Ots28_11075348 | 0.204 | 0.555 | 0.343 | 0.26897361 | 0.96331593 | 0.15538387 |
| Ots28_11077172 | 0.204 | 0.555 | 0.343 | 0.26897361 | 0.96331593 | 0.15538387 |
| Ots28_11073102 | 0.204 | 0.577 | 0.373 | 0.26897361 | 0.96331593 | 0.15538387 |
| Ots28_11078636 | 0.204 | 0.577 | 0.373 | 0.28048606 | 0.96331593 | 0.15538387 |
| Ots28_11207428 | 0.204 | 0.555 | 0.688 | 0.52145393 | 0.96331593 | 0.99573803 |

**Figure S1.** Map of Columbia River Basin with four distinct collection locations that represent three major phylogenetic lineages of Chinook Salmon in North America; 1, Cowlitz Hatchery (Lower Columbia lineage); 2, Prosser Hatchery (fall collection of Interior ocean-type lineage); 3, Wells Hatchery (summer collection of Interior ocean-type lineage); and 4, Johnson Creek Weir (Interior stream-type lineage).

**Figure S2**. Density plots for the number of adult offspring assigned to parents from return year 2010 and 2011.


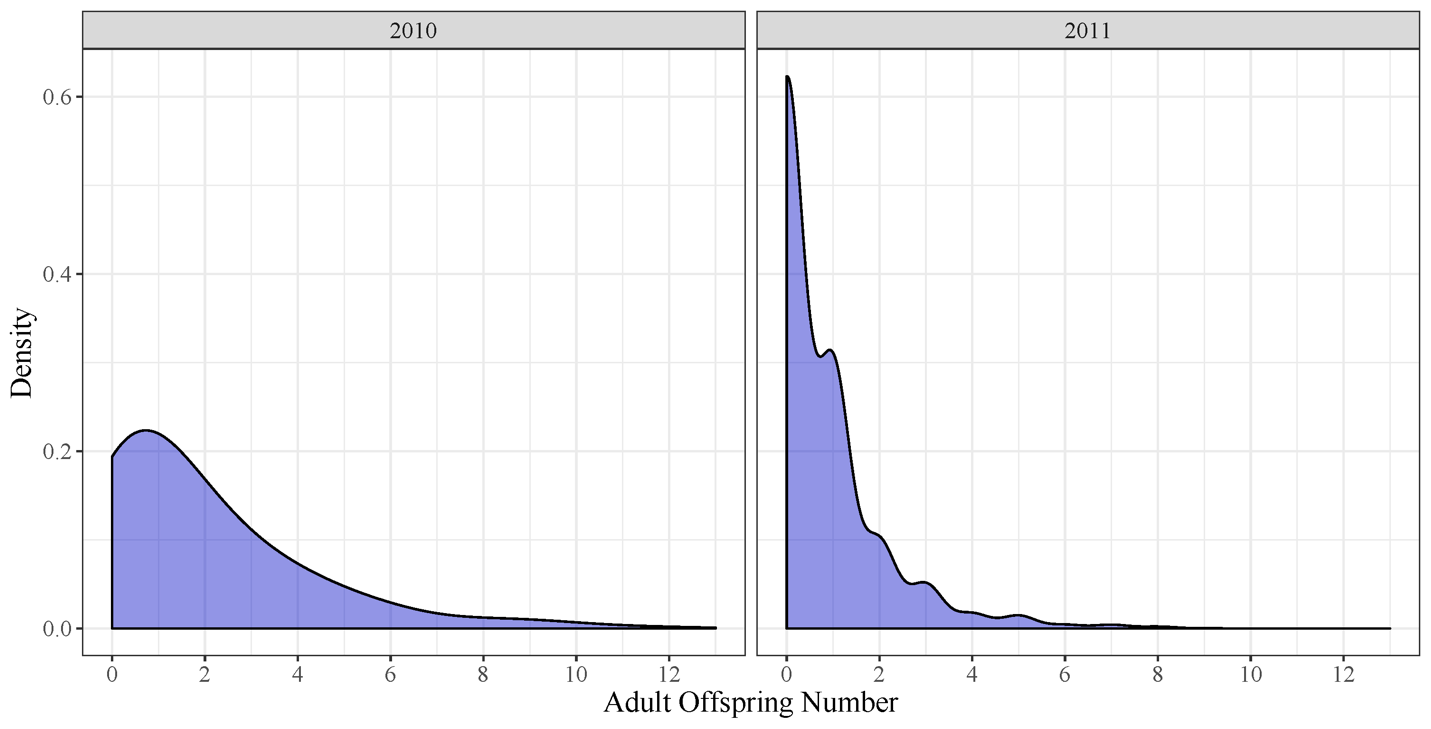


**Figure S3**. PCA of 33 chromosome 28 markers for the Interior stream-type population separated by year of return. Ellipses represent 95% confidence levels.

**Figure S4.** Significant SNP from BLINK as implemented in GAPIT for the Lower Columbia lineage and relationship to early versus late arrival timing phenotypes. Density plots represent the most significant SNP from the GAPIT association results.

**
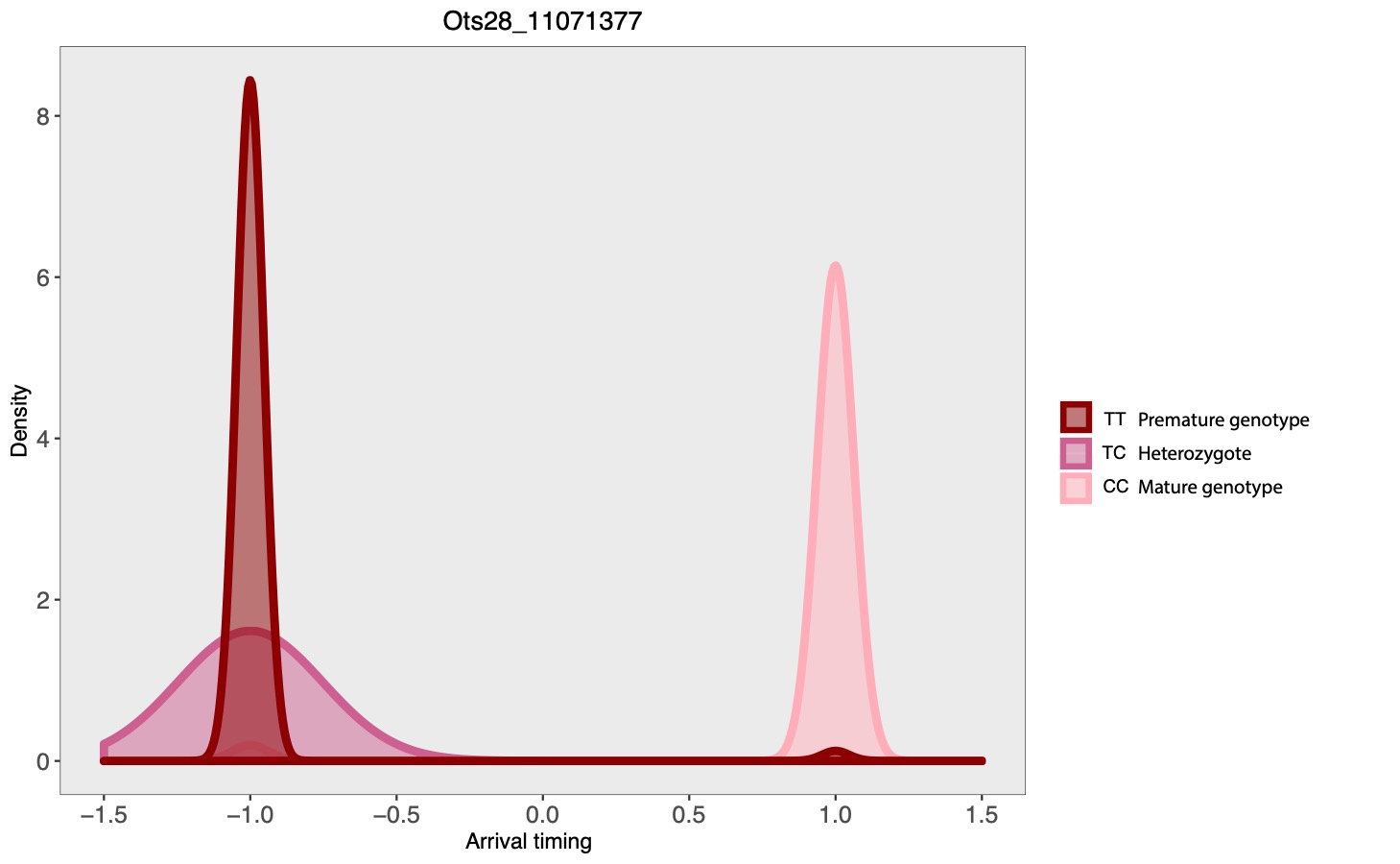
**

**Figure S5.** Significant SNPs from BLINK as implemented in GAPIT for the Interior ocean-type lineage and relationship to early versus late arrival timing phenotypes. Density plots represent the most significant SNP from the GAPIT association results.

**
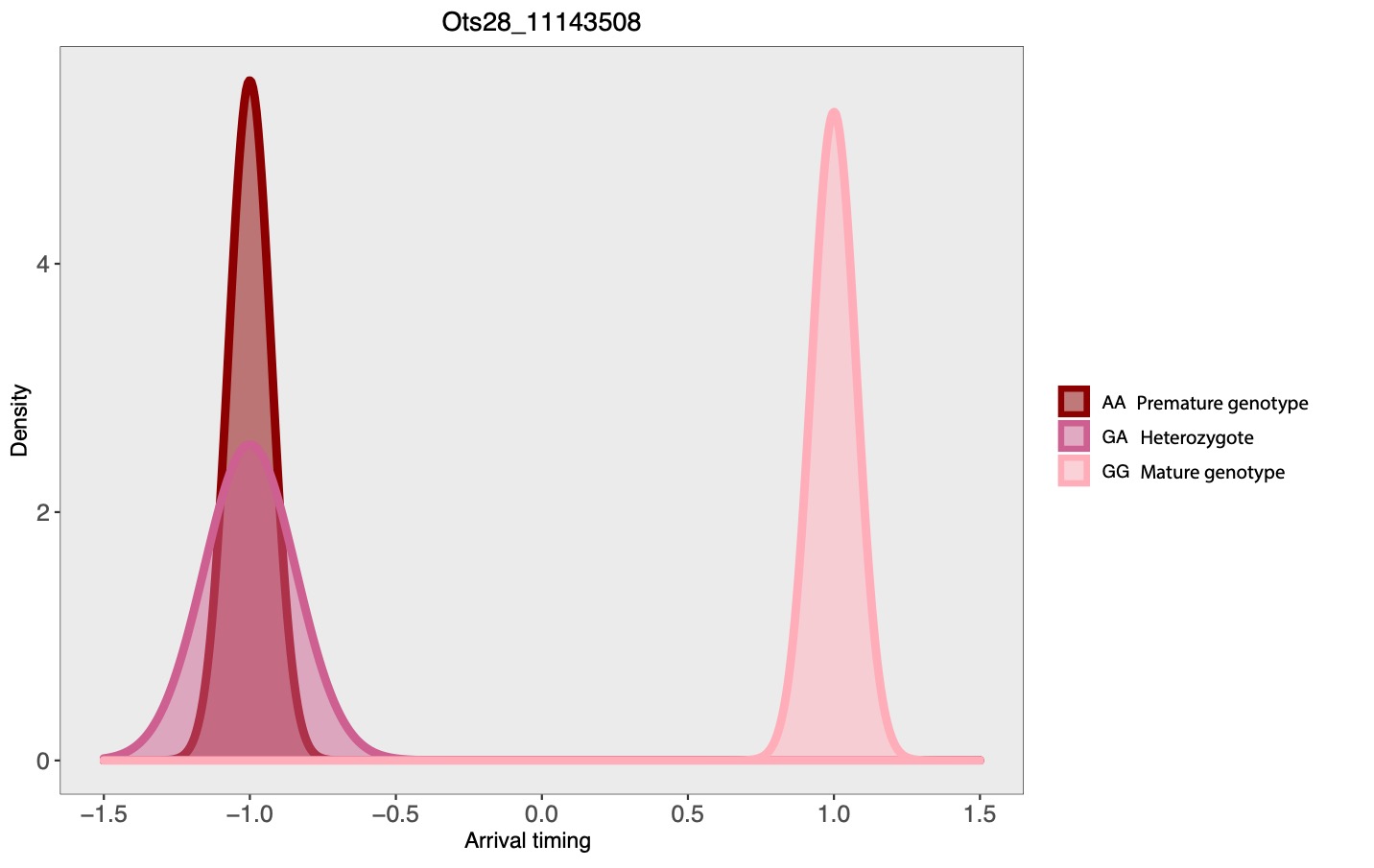
**

**Figure S6.** Significant SNPs from BLINK as implemented in GAPIT for the Interior stream-type lineage and relationship to early versus late arrival timing phenotypes. Density plots represent the most significant two SNPs (A and B) from the GAPIT association results separated by return year.


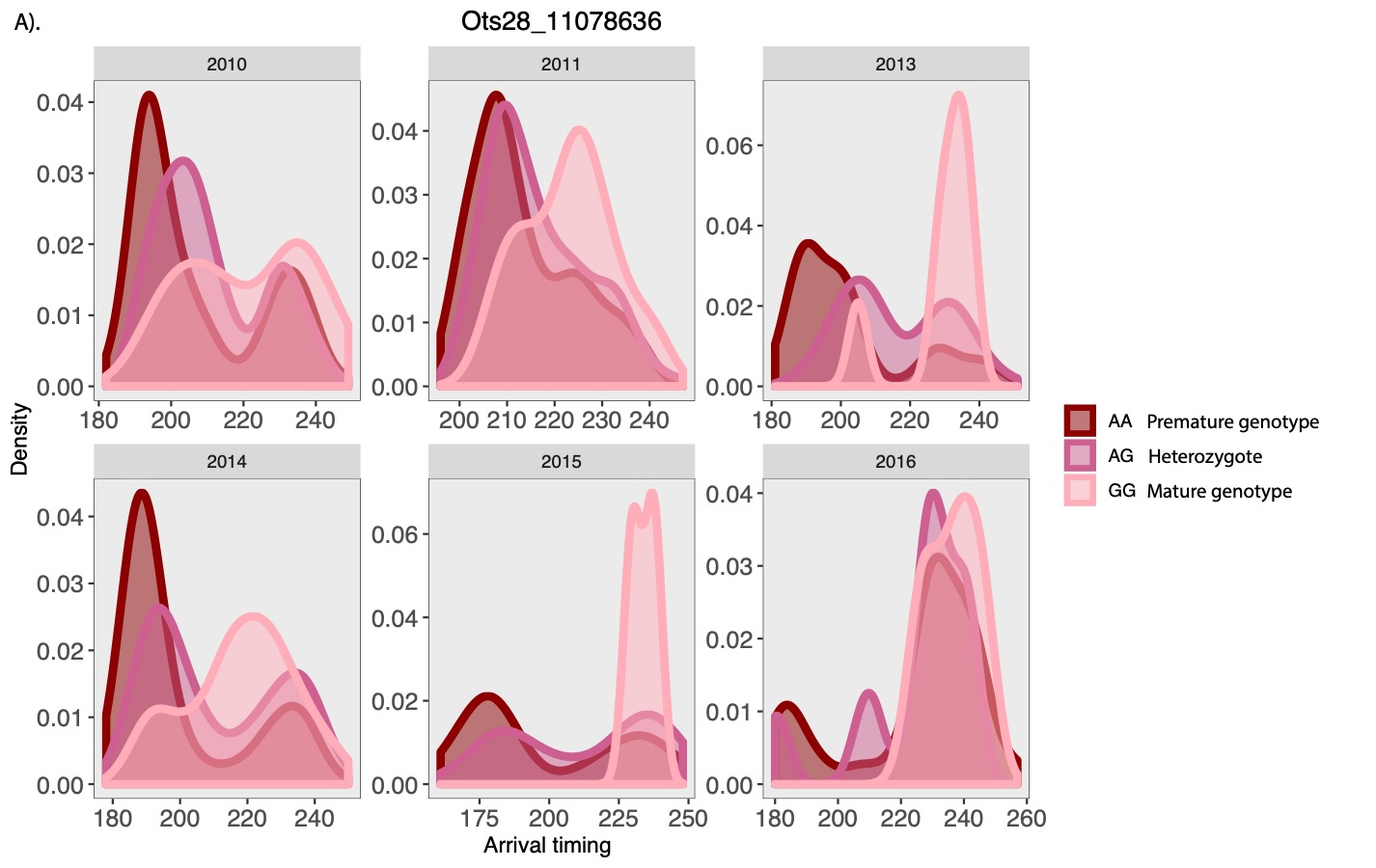


**
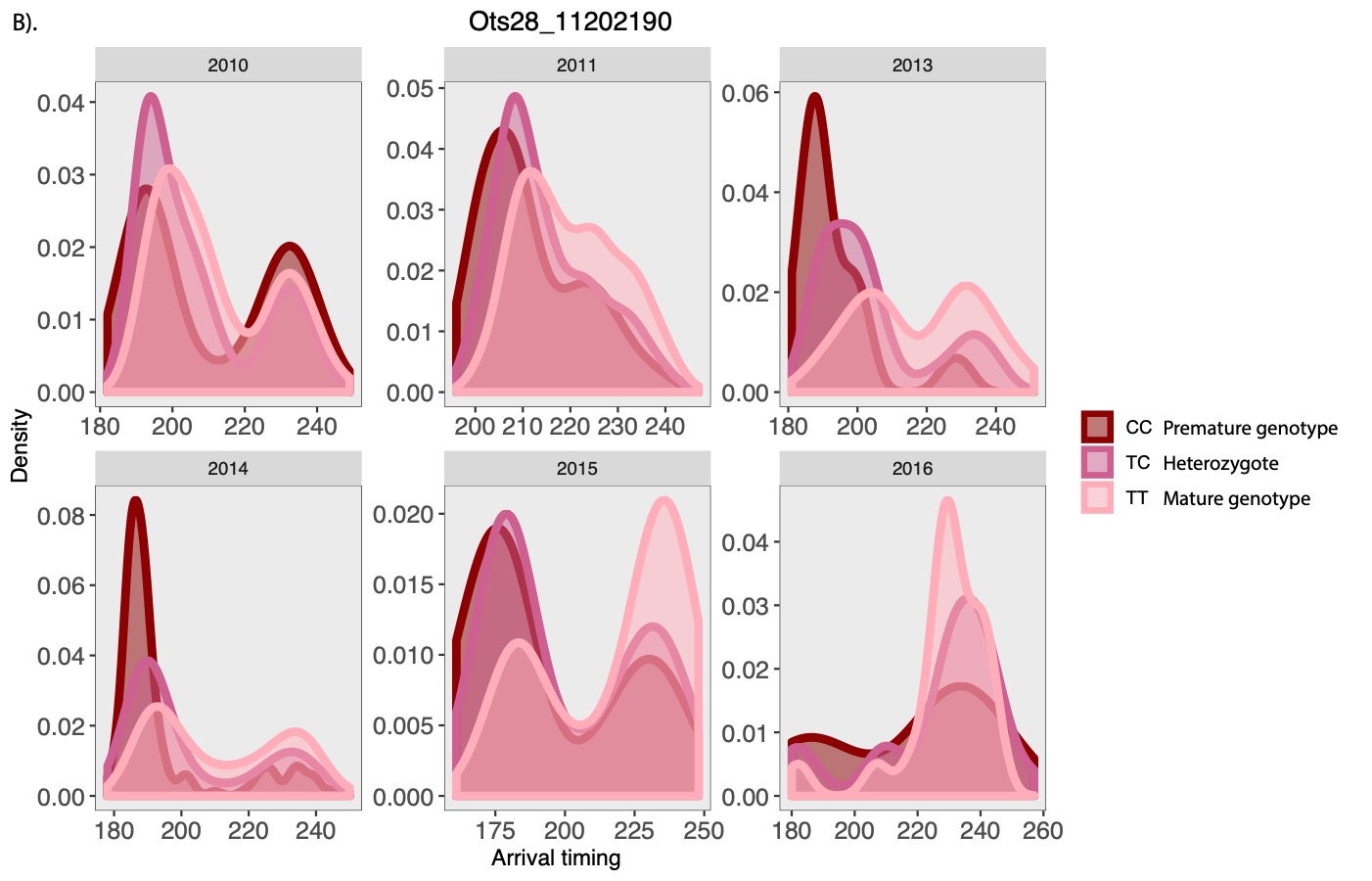
**

**Figure S7**. Scatter plot of arrival timing and fitness separated by sex for the Interior stream-type population. Cut-off used for early versus late arrival to spawning grounds category designation was ordinal day 216 (August 4^th^).

**References**

Narum, S. R., Di Genova, A., Micheletti, S. J., & Maass, A. (2018). Genomic variation underlying complex life-history traits revealed by genome sequencing in Chinook salmon. Proc. R. Soc. B, 285(1883), 20180935.
